# Dietary Insulinogenic Amino Acid Restriction Improves Glucose Metabolism in a Neonatal Piglet Model

**DOI:** 10.3390/nu17101675

**Published:** 2025-05-15

**Authors:** Matthew W. Gorton, Parniyan Goodarzi, Xia Lei, Michael Anderson, Mohammad Habibi, Nedra Wilson, Adel Pezeshki

**Affiliations:** 1 Department of Animal and Food Sciences, Oklahoma State University, Stillwater, OK 74078, USA; matthew.gorton@okstate.edu (M.W.G.); goodarzi@wustl.edu (P.G.); habibi@wustl.edu (M.H.); 2 Department of Biochemistry and Molecular Biology, Oklahoma State University, Stillwater, OK 74078, USA; xia.lei@okstate.edu; 3 Department of Anatomy and Cell Biology, Oklahoma State University Center for Health Sciences, Tulsa, OK 74107, USA; michael.b.anderson@okstate.edu (M.A.); nedra.wilson@okstate.edu (N.W.)

**Keywords:** insulinogenic amino acids, glucose metabolism, insulin sensitivity, FGF-21 signaling, neonatal pigs

## Abstract

**Background:** Dietary consumption of insulinogenic amino acids (IAA) is known to contribute to the development of insulin resistance. It remains to be studied whether dietary IAA restriction improves glucose metabolism and insulin sensitivity and whether this improvement is related to alterations in glucose metabolism in peripheral tissues. The objective of this study was to examine the effect of IAA restriction on glucose metabolism in a piglet model. **Methods:** Following the acclimation period, thirty-two seven-day-old male piglets were randomly assigned into one of three groups for three weeks as follows (*n* = 10–11/group): (1) NR (control): basal diet without IAA restriction; (2) R50: basal diet with IAA restricted by 50%; (3) R75: basal diet with IAA restricted by 75%. IAA were alanine (Ala), arginine (Arg), isoleucine (Ile), leucine (Leu), lysine (Lys), threonine (Thr), phenylalanine (Phe), and valine (Val) as suggested by previous studies. Thermal images, body weight, and growth parameters were recorded weekly, oral glucose tolerance tests were performed on week 2 of the study, and blood and tissue samples were collected on week 3 after a meal test. **Results:** R75 improved glucose tolerance and, together with R50, reduced blood insulin concentration and homeostatic model assessment for insulin resistance (HOMA-IR) value, which is suggestive of improved insulin sensitivity following IAA restriction. R75 increased thermal radiation and decreased adipocyte number in white adipose tissue (WAT). R75 had a greater transcript of glucose transporter 1 (GLUT1), phosphofructokinase, liver type (PFKL), and pyruvate kinase, liver, and RBC (PKLR) in the liver and glucokinase (GCK) in WAT indicating a higher uptake of glucose in the liver and greater glycolysis in both liver and WAT. R75 increased the mRNA abundance of insulin receptor substrate 1 (IRS1) and protein kinase B (AKT1) in skeletal muscle suggestive of enhanced insulin signaling. Further, R75 had a higher mRNA of fibroblast growth factor 21 (FGF-21) in both the liver and hypothalamus and its upstream molecules such as activating transcription factor 4 (ATF4) and inhibin subunit beta E (INHBE) which may contribute to increased energy expenditure and improved glucose tolerance during IAA restriction. **Conclusions:** IAA restriction improves glucose tolerance and insulin sensitivity in piglets while not reducing body weight, likely through improved hepatic glycolysis and insulin signaling in skeletal muscle, and induced FGF-21 signaling in both the liver and hypothalamus.

## 1. Introduction

The prevalence of obesity and type 2 diabetes (T2D) continues to rise in children globally [1,2] and is considered a growing public health problem in the United States [3,4,5]. According to the CDC, the rate of new cases of T2D in children aged < 20 years had a 4.8% increase per year between 2002 and 2015 in the US [6,7]. In 2018, 26.9 million people were diagnosed with diabetes in the US, of which 210,000 were children and adolescents younger than age 20 years [8]. The incidence and prevalence of T2D in US adolescents is projected to increase by more than four times in the following decades [9].

According to the early protein hypothesis, increased protein intake in early life is associated with later adverse health outcomes [10,11,12,13]. An elevated protein intake triggers insulin-like growth factor 1 (IGF-I) and insulin secretion, which increase growth, weight gain, and adiposity [14,15]. The infant formula with protein levels closer to what is naturally found in human breastmilk has been found to not significantly alter the child’s BMI or weight-for-age [16,17,18]. Due to limited and complex data available on the advantages of low-protein formula and the beneficial effects of amino acids (AA) for maintaining the body’s protein stores [11,19], it is premature to prescribe low-protein formula to infants [20]. Thus, to minimize the outcome of increased early protein intake on chronic diseases later in life, the development of novel nutritional interventions is crucial.

A group of AA including leucine (Leu), isoleucine (Ile), valine (Val), lysine (Lys), arginine (Arg), threonine (Thr), phenylalanine (Phe), and alanine (Ala) have been linked to a rapid increase in insulin secretion leading to hyperinsulinemia, which could eventually increase the risk of insulin resistance [21,22,23,24,25,26,27]. The elevated concentration of these AA may induce the secretion of IGF-I and the expression of mTOR, which contribute to the development of insulin resistance [12,28]. Previous studies have shown that restriction of branched-chain amino acids (BCAA, i.e., Leu, Ile, and Val) improves insulin sensitivity and key metabolic pathways regulating glucose homeostasis and lipid metabolism in murine models [29,30,31,32,33,34]. Whether dietary IAA restriction in early life improves metabolic health in later stages in humans is poorly understood. The effect of IAA restriction during the neonatal period on adolescent obesity and body composition is largely unknown. Further, less is known about the impact of restriction of all eight IAA on glycemic control and glucose and lipid metabolism in peripheral tissues.

There are significant limitations in using rodents as a neonatal nutritional model due to their short nursery period and challenges in feeding their offspring with formula right after birth. Neonatal pigs with a relatively long suckling period have been extensively used as a model for the human infant’s nutrition [35,36]. Further, pigs have been used as a model for studying various physiological functions of humans, such as AA metabolism and metabolic complications such as T2D [37,38]. Therefore, in this study, we used neonatal pigs as a translational animal model for human infants’ nutrition in early life. As such, we examined the impact of two levels of IAA restriction on glucose metabolism in neonatal pigs.

## 2. Materials and Methods

### 2.1. Animals and Housing

All experimental procedures used for this study were in accordance with the FASS Guide for Care and Use of Agricultural Animals in Research and Teaching [39] and were approved by the Institutional Animal Care and Use Committee of Oklahoma State University (Animal Care and Use Protocol-IACUC-19-71). Thirty-two seven-day-old male piglets (mean body weight of 2.02 ± 0.44 kg) were selected from four sows (Yorkshire, parity: 3, litter size: 13–14; Oklahoma State University). Each piglet received an intramuscular injection of iron hydrogenated dextran complex (Sparhawk Laboratories, Inc Lenexa, KS, USA, 100 mg/mL) at 100 mg/kg body weight and antibiotic (EXCEDE^®^ for Swine, Zoetis, Kalamazoo, MI, USA, 100 mg/mL) at 5 mg/kg body weight on day three postpartum. All piglets were housed individually in plastic floor pens (0.86 × 0.79 × 0.81 m), with a heat mat and milk replacer feeder. The lighting protocol was based on a 16:8 light–dark cycle with the lights on at 0600 and off at 2200 throughout the study [40]. Room temperature was maintained at 30 °C, 29 °C, and 28 °C during weeks 1, 2, and 3 of the study, respectively [41].

### 2.2. Experimental Design and Diets

**Figure 1:**
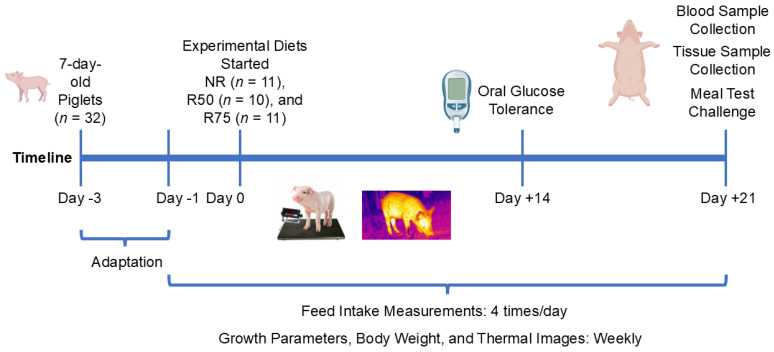
Schematic of study design. Neonatal piglets were randomly assigned to one of three dietary treatments, including NR: basal diet without restricted insulinogenic amino acid (IAA); R50: basal diet with 50% restricted IAA; R75: basal diet with 75% restricted IAA, for 3 weeks. Oral glucose tolerance test (OGTT) (1 g glucose/kg body weight) was performed on day 14 following an overnight fasting. Feed intake was measured 4 times/day, and body weights, thermal images, and growth parameters were recorded weekly. In week 3, following an overnight fast, all piglets were allowed to consume their respective diet for 30 min, and blood samples were collected at 0, 30, 60, 90, and 120 min. All animals were sacrificed, and tissue samples were collected 120 min following the meal test.

Seven-day-old piglets were group-housed and acclimated to the environment for 3 days while receiving the control diet as described below. Following the acclimation period, piglets were weight-matched and randomly assigned into one of three groups for three weeks as follows (*n* = 10–11/group; 2–3 piglets for each group were obtained from each of the four sows): (1) NR (control): basal diet without IAA restriction (2.28 ± 0.40 kg); (2) R50: basal diet with IAA restricted by 50% (2.28 ± 0.51 kg); (3) R75: basal diet with IAA restricted by 75% (2.29 ± 0.53 kg). IAA were Ala, Arg, Ile, Leu, Lys, Thr, Phe, and Val as suggested by previous studies [24,42,43]. This group of AA has been linked to hyperinsulinemia and increased risk of insulin resistance [21,22,23,24,25,26,27]. To the best of our knowledge, no previous study has looked at the synergistic or additive effects of restriction of eight selected amino acids on glucose metabolism, although certain combinations of the selected IAA, such as Lys, Thr, Leu, Ile, and Val, were reported to be correlated with hyperinsulinemia. Since the majority of studies used restrictions of individual IAA or BCAA at rates of 30–95% and found the positive effects of AA restriction at rates higher than 50% [44,45,46,47], we decided to apply a 50% or 75% restriction of IAA in this study. The experimental timeline is illustrated in Figure 1.

The basal milk replacer powder (NR) and R50 and R75 diets were formulated according to nutrient requirements for suckling piglets [48,49] (Table 1). For the NR basal diet, the level of essential AA was maintained at the concentration of those AA that are naturally found in sows’ milk [50]. All diets were kept isocaloric and isonitrogenous via supplementation of dextrose and L-glutamic acid, respectively. Other than IAA, dextrose, and L-glutamic acid, the rest of the dietary ingredients were kept constant across all diets. For the preparation of the liquid milk replacers, 1 kg of the respective diets powder (97% dry matter (DM)) were reconstituted with 4.2 L of warm water (55 °C) to achieve the 18.6% DM in milk replacers similar to what is naturally found in sow’s milk [50]. Liquid milk replacers were prepared daily and stored at 4 °C. Prior to feeding, milk replacers were warmed up to 40 °C via a water bath and then transferred to milk bottles for each piglet. The milk bottle for each feeder was placed on an electric hot plate stirrer to keep the milk replacer homogenous and at 40 °C (±1) throughout the day to maintain palatability. Each piglet was fed 4 times per day at 0600, 1200, 1800, and 0000 and had ad libitum access to the milk replacer during each meal throughout the experiment by receiving a minimum of 60 g DM/kg BW/day of milk replacer [50,51].

### 2.3. Feed Intake and Growth Measurements

The 24 h milk replacer intake was calculated by summing up the feed intake measured at 0600, 1200, 1800, and 0000. Body weight, heart girth, body length, and wither size were recorded weekly. Average daily gain (ADG), average dry matter intake (ADMI), cumulative dry matter intake (CDMI), average daily protein intake (ADPI), gain-to-feed ratio (G:F), and gain-to-protein ratio (G:P) were calculated accordingly.

### 2.4. Thermal Images

Using a FLIR C2 compact thermal camera (accuracy of ±2 °C; focal length of 1.54 mm; emissivity coefficient of 0.96), thermal images were captured for all pigs weekly (FLIR Systems, Boston, MA, USA). The camera was consistently positioned roughly 1 m above all animals.

### 2.5. Oral Glucose Tolerance Test

Oral glucose tolerance tests (OGTT) were performed on day 14 of the study. Briefly, following an overnight fast (8 h), basal blood glucose was recorded via a handheld glucometer. Each piglet was then administered orally with 1 g dextrose (50% in saline)/kg BW [52], followed by blood glucose measurements at 30, 60, 90, and 120 min from the medial auricular vein via needle pricking.

### 2.6. Feed Samples

Feed samples (~1 kg) were collected from each feed bag and pooled for each diet during diet preparations. All feed samples were stored at −20 °C until chemical composition analysis as described below.

### 2.7. Meal Test and Blood and Tissue Collection

Following an overnight fast (8 h) on day 21, each piglet was allowed to consume their respective diet for 30 min. Following the meal test, blood samples were collected at baseline and then at, 60, 90, and 120 min post-meal from the jugular vein into 10 mL serum tubes (Monoject™, Covidien Mansfield, MA, USA) and 3 mL heparin-containing plasma tubes (BD Vacutainer^®^, Franklin Lakes, NJ, USA). Blood glucose concentrations were recorded at the above timepoints via a handheld glucometer. All blood samples were centrifuged at 3000× *g* for 15 min at 4 °C. Plasma and serum were separated and stored at −80 °C for future analysis. Using CO_2_ asphyxiation, all piglets were sacrificed 120 min after the meal test. Immediately after sacrifice, tissue samples, including liver, skeletal muscle, hypothalamus, and white adipose tissue (WAT), were collected, rinsed in distilled water, snap-frozen in liquid nitrogen, and stored at −80 °C for later analysis [53]. Further, additional WAT samples were collected, rinsed in distilled water, and fixed in 10% formaldehyde.

### 2.8. Diets Proximate and Amino Acid Analysis

Chemical composition of diets (i.e., dry matter, crude protein, phosphorus, and calcium) was analyzed by Servi-Tech laboratory (Dodge City, KS, USA) [53,54,55,56]. The AA profile of the diets was analyzed at Agricultural Experiment Station Chemical Laboratories, University of Missouri (Columbia, MO, USA) as we previously described [41] (Table 2).

### 2.9. Thermal Radiation Analysis

FLIR camera software (FLIR Research Studio software Version 5.13.18031.2002) free drawing tool, was used to draw a rectangular region of interest encompassing the whole back (shoulders to rump) of each piglet to determine the dorsal surface body average temperature. Thermal radiation related to heat loss was calculated as we previously described [57,58,59,60].

### 2.10. Plasma Insulin

A porcine ELISA kit was used to determine plasma insulin concentration, according to the manufacturer’s specifications (Mercodia Porcine Insulin ELISA, Mercodia AB; Uppsala, Sweden). Optical density was measured at 450 nm wavelength using an Epoch microplate spectrophotometer (BioTek^®^ Instruments, Inc., Highland Park, VT, USA). The detection range of the ELISA assay was 2.3–173 mU/L with antibodies specific to mature insulin. The intra-assay coefficient of variation was 3%.

### 2.11. Insulin Sensitivity Calculations

Homeostatic model assessment for insulin resistance (HOMA-IR) was calculated as an insulin resistance indicator using the following equation: I0 × G0/22.5, with I0 being fasting plasma insulin (μU/mL) and G0 being fasting plasma glucose (mM) [61]. The quantitative insulin sensitivity check index (QUICKI) was calculated as 1/[log(I0) + log(G0)], where I0 is fasting insulin (μU/mL) and G0 is fasting glucose (mg/dL) [62].

### 2.12. H&E Staining and Adipocyte Measurements

Fixed inguinal WAT samples in 10% formaldehyde were coated in paraffin, cut into 8-micrometer-thick sections, and stained with hematoxylin and eosin. Sections were then imaged using an EVOS M5000 fluorescence microscope imaging system (Thermo Fisher, Waltham, MA, USA). The analysis for surface area and number of adipocytes in captured images was performed using Fiji/ImageJ (29 June 2023, https://imageJ.net/Fiji/Downloads Version 1.54f) as previously described [63].

### 2.13. RNA Isolation and RT-qPCR

RNA was isolated from liver, skeletal muscle, WAT, and hypothalamus. RT-qPCR was performed on glucose, lipid, and glycogen metabolism genes (Supplementary Table S1) following our published procedures [64,65,66]. Briefly, isolated RNA concentration and 260:280 nm absorbance were measured by a NanoDrop ND-1000 spectrophotometer (Thermo Fisher, Waltham, MA, USA). A T100^TM^ Thermal Cycler (Bio-Rad, Hercules, CA, USA) was used to synthesize the complementary DNA. The mRNA abundance of the target genes was measured using a CFX96 real-time PCR detection system (Bio-Rad, Hercules, CA, USA) via real-time quantitative PCR (qPCR) using the primers obtained from previous publications or designed in-house [67,68,69,70,71,72,73,74,75,76,77,78,79,80,81,82,83,84,85,86,87,88,89,90,91,92,93,94]. The relative mRNA abundance of the target genes was calculated using the 2^−∆∆CT^ method using Beta-actin (β-Actin) as a housekeeping gene.

### 2.14. Immunoblot

Western blot was conducted in the liver for fibroblast growth factor 21 (FGF21) (Supplementary Table S2), as previously described in [95,96]. The protein bands were visualized, and images were captured using a ChemiDoc XR imaging system (Bio-Rad Laboratories Inc., Hercules, CA, USA), followed by densitometry calculations using ImageLab software (version 6.0.1, Bio-Rad Laboratories Inc., CA, USA). Glycaldhyde-3-phosphate dehydrogenase (GAPDH) was used as a loading control to determine the relative protein abundance in the samples.

### 2.15. Statistical Analysis

Before statistical analysis, an outlier test based on the Interquartile Rule was performed on all data in SPSS (IBM SPSS Statistical Version 26, Armonk, NY, USA). Data were then checked for normality, and if the data distribution was not normal, they were normalized using an inverse distribution function (IDF-normal; IBM SPSS Statistical Version 26, Armonk, NY, USA). Growth parameters, including heart girth, body length, wither size, final body weight, etc., were analyzed via GLM univariate procedure, followed by Dunnett’s post hoc test (IBM SPSS Statistical Version 26, Armonk, NY, USA) as we previously described [41]. Blood glucose and thermal radiation data were analyzed with the mixed procedure using the pen as a random variable and time, diet, and the interaction of time and diet as fixed variables, similar to our previous research [54,55,64]. Blood glucose concentration following the meal test was adjusted with DMI as a covariate via GLM univariate procedure, followed by Sidak post hoc test (IBM SPSS Statistical Version 26, Armonk, NY, USA). *p* ≤ 0.05 was considered significant and 0.05 < *p* ≤ 0.10 was interpreted as a trend for significance.

## 3. Results

### 3.1. Body Weight, Growth Measurements, and Feed Intake

Body weight, growth parameters, and ADMI were not different among groups when compared to NR (Supplementary Table S3). The CDMI tended to be significant (*p* value = 0.07) among groups, and post hoc analysis showed a significantly higher (*p* < 0.05) CDMI for R75 compared to NR (Supplementary Table S3). Although the overall *p*-value for body length was not significant (*p* > 0.05), R75 tended to have a lower body length than NR (*p* = 0.07) (Supplementary Table S3).

### 3.2. Glucose Tolerance, Plasma Insulin, and Insulin Sensitivity Indices

**Figure 2:**
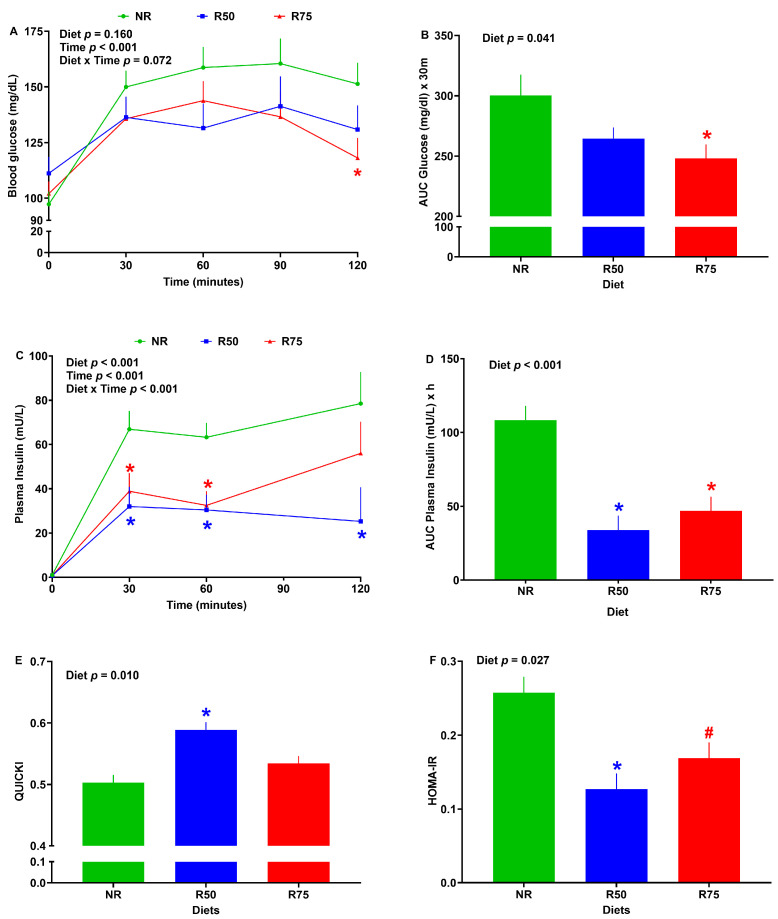
Effect of insulinogenic amino acids (IAA) restriction on glucose tolerance, insulin concentration, and insulin sensitivity measures in neonatal pigs. (**A**,**B**) Blood glucose and area under the curve (AUC) for glucose after oral glucose tolerance test, (**C**,**D**) plasma insulin and AUC for plasma insulin after meal test, (**E**) quantitative insulin sensitivity check index (QUICKI), (**F**) homeostatic model assessment for insulin resistance (HOMA-IR). NR: basal diet without restricted IAA; R50: basal diet with 50% restricted IAA; R75: basal diet with 75% restricted IAA. The values are the means ± SE. *n* = 6–10 for NR, *n* = 4–8 for R50, and *n* = 6–11 for R75. ^#^ *p* ≤ 0.1 vs. NR. * *p* ≤ 0.05 vs. NR.

Following the OGTT, no significant differences in blood glucose were observed among groups at 0, 30, 60, and 90 min (Figure 2A). However, R75 did have a 22% reduction in blood glucose when compared to NR at 120 min (Figure 2A). Further, R75 had a 17% lower blood glucose AUC than NR following OGTT (Figure 2B). No significant differences in blood glucose were observed among groups following the meal test at the end of the study when DMI during the meal test was used as a covariate for analysis of blood glucose (Supplementary Figure S1A,B).

There was no difference in baseline plasma insulin concentrations across groups (Figure 2C). Plasma insulin concentration was decreased for R50 and R75 compared to NR until 120 and 60 min after starting the meal challenge, respectively (Figure 2C). Both R50 and R75 groups had a lower plasma insulin AUC than NR following the meal test (Figure 2D). In comparison to NR, R50 had higher QUICKI and lower HOMA-IR values. Additionally, R75 tended to have lower HOMA-IR scores than NR (Figure 2E,F).

### 3.3. Thermal Radiation

**Figure 3:**
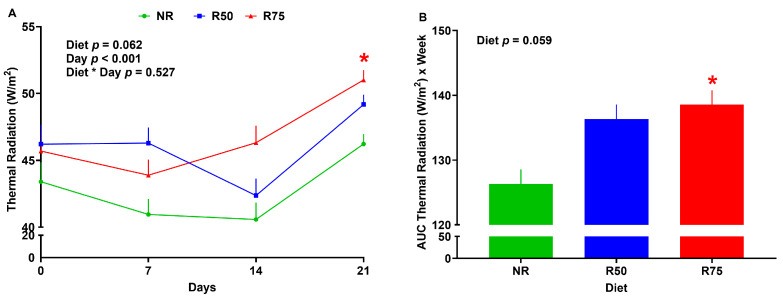
Effect of insulinogenic amino acid (IAA) restriction on thermal radiation in neonatal pigs. (**A**) thermal radiation, (**B**) area under the curve (AUC) for thermal radiation. NR: basal diet without restricted IAA; R50: basal diet with 50% restricted IAA; R75: basal diet with 75% restricted IAA. The values are the means ± SE. *n* = 6–10 for NR., *n* = 6–10 for R50, and *n* = 9–11 for R75. * *p* ≤ 0.05 vs. NR.

Compared to NR, there was no difference in thermal radiation and the AUC for thermal radiation for the R50 treatment (Figure 3A,B). The R75 group increased thermal radiation in week 3, and had a higher AUC for thermal radiation than NR (Figure 3A,B).

### 3.4. Size and Number of Adipocytes in White Adipose Tissue

**Figure 4:**
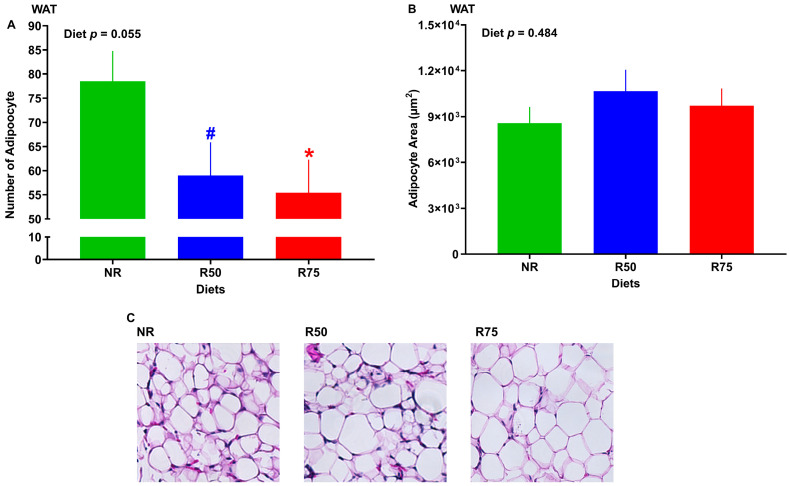
Effects of insulinogenic amino acid (IAA) restriction on the quantity and size of adipocytes in inguinal adipose tissue of neonatal pigs. (**A**) Number of adipocytes, (**B**) adipocyte surface area, and (**C**) representative images of hematoxylin-and-eosin-stained sections (40× magnification). NR: basal diet without restricted IAA; R50: basal diet with 50% restricted IAA; R75: basal diet with 75% restricted IAA. The values are the means ± SE. *n* = 7 for NR, *n = 5* for R50, and *n = 6* for R75. ^#^ *p* ≤ 0.1 vs. NR. * *p* ≤ 0.05 vs. NR.

Adipose tissue histology showed that adipocyte numbers were significantly lower in R75 and tended to be lower in R50 groups compared to NR. However, there were no significant differences in adipocyte size when compared to NR (Figure 4A–C).

### 3.5. mRNA Abundance of Genes Involved in Glucose Transport and Metabolism, Glycogen Metabolism, and Insulin Signaling

**Figure 5:**
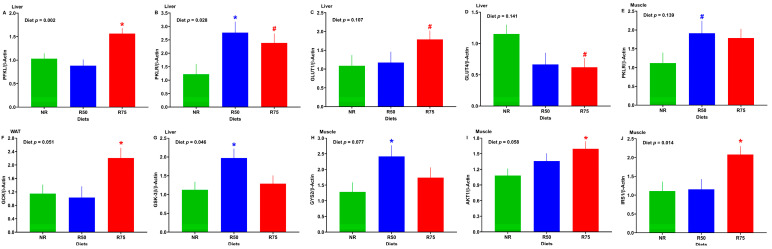
Effect of insulinogenic amino acid (IAA) restriction on mRNA abundance of genes involved in glucose transport and metabolism, glycogen metabolism, and insulin signaling in the liver, white adipose tissue (WAT), and skeletal muscle of neonatal pigs. (**A**) Liver phosphofructokinase, liver type (PFKL), (**B**) liver pyruvate kinase, liver and RBC (PKLR), (**C**) liver glucose transporter 1 (GLUT1), (**D**) liver glucose transporter 4 (GLUT4), (**E**) skeletal muscle PKLR, (**F**) WAT glucokinase (GCK), (**G**) liver glycogen synthase kinase 3β (GSK-3β), (**H**) skeletal muscle glycogen synthase 2 (GYS2), (**I**) skeletal muscle protein kinase B (AKT1), and (**J**) skeletal muscle insulin receptor substrate 1 (IRS1). NR: basal diet without restricted IAA; R50: basal diet with 50% restricted IAA; R75: basal diet with 75% restricted IAA. The values are the means ± SE. *n* = 7–8 for NR, *n* = 5–7 for R50, and *n* = 5–9 for R75. ^#^ *p* ≤ 0.1 vs. NR. * *p* ≤ 0.05 vs. NR.

While the mRNA abundance of hepatic pyruvate kinase, liver, and RBC (PKLR) was higher for both R50 and R75 (*p* < 0.1), the transcript for phosphofructokinase, liver type (PFKL), and glucose transporter 1 (GLUT1) (*p* < 0.1) were only greater in R75 compared to NR (Figure 5A–C). The mRNA abundance of liver glucose transporter 4 (GLUT4) tended to be diminished for the R75 treatment group (Figure 5D). Skeletal muscle PKLR tended to be greater in R50, and WAT glucokinase (GCK) was higher in R75 when compared to NR (Figure 5E,F). The R50 group had a greater mRNA abundance of glycogen synthase kinase 3β (GSK-3β) and glycogen synthase 2 (GYS2) in the liver and skeletal muscle, respectively (Figure 5G,H). The R75 group had a greater transcript of protein kinase B (AKT1) and insulin receptor substrate 1 (IRS1) in skeletal muscle relative to NR (Figure 5I,J). No significant differences were found among treatment groups for the mRNA abundance of other genes involved in glucose transport and metabolism, glycogen metabolism, and insulin signaling (Supplementary Figures S2–S4).

### 3.6. mRNA Abundance of Genes Involved in Lipid Metabolism

**Figure 6:**
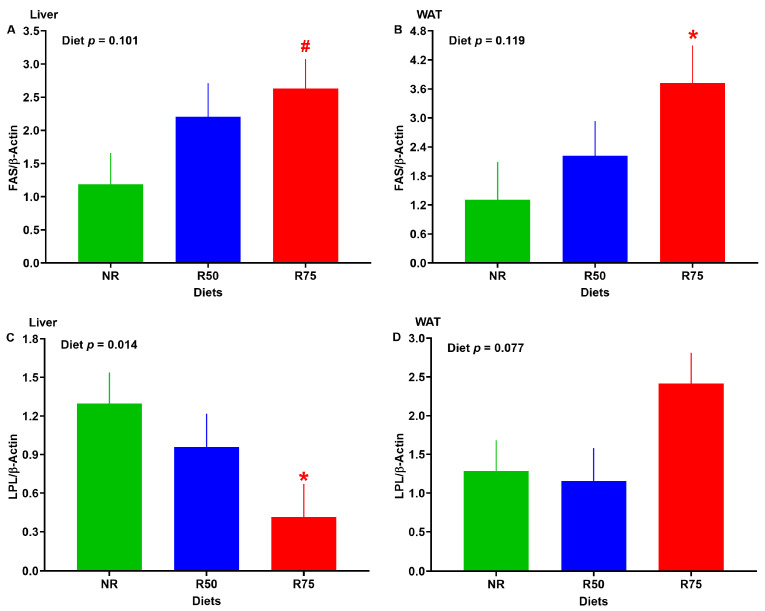
Effect of insulinogenic amino acid (IAA) restriction on mRNA abundance of genes involved in lipid metabolism in the liver and white adipose tissue (WAT) of neonatal pigs. (**A**) liver fatty acid synthase (FAS), (**B**) WAT FAS, (**C**) liver lipoprotein lipase (LPL), and (**D**) WAT LPL. NR: basal diet without restricted IAA; R50: basal diet with 50% restricted IAA; R75: basal diet with 75% restricted IAA. The values are the means ± SE. *n* = 7–8 for NR, *n* = 5–7 for R50, and *n* = 5–9 for R75. ^#^ *p* ≤ 0.1 vs. NR. * *p* ≤ 0.05 vs. NR.

The mRNA abundance of hepatic fatty acid synthase (FAS) tended to be greater, with lipoprotein lipase (LPL) significantly decreased in R75 when compared to NR (Figure 6A,C). In WAT, the mRNA abundance of FAS was significantly increased in R75 (Figure 6B). The overall *p*-value for mRNA abundance of LPL in WAT tended to be significant, with R75 having the highest expression among groups (Figure 6D). No significant difference was seen among groups for other key genes involved in lipid metabolism (Supplementary Figure S5).

### 3.7. mRNA Abundance of Genes Associated with FGF-21 Pathway or FGF-21 Protein Expression

**Figure 7:**
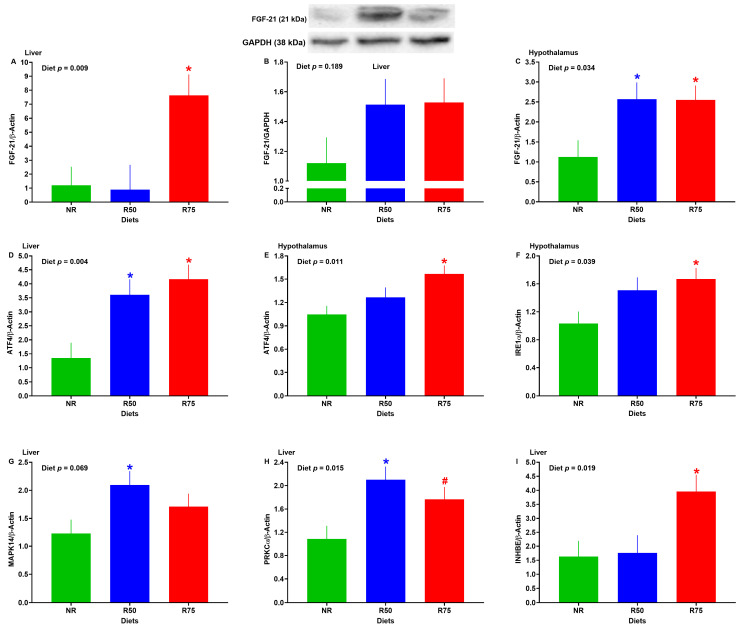
Effect of insulinogenic amino acid (IAA) restriction on mRNA abundance of genes associated with fibroblast growth factor 21 (FGF-21) pathway or FGF-21 protein expression in the liver and hypothalamus of neonatal pigs. (**A**,**B**) Liver FGF-21, (**C**) hypothalamus FGF-21, (**D**) liver activating transcription factor 4 (ATF4), (**E**) hypothalamus ATF4, (**F**) hypothalamus inositol-requiring enzyme type 1α (IRE1α), (**G**) liver p38α mitogen-activated protein kinase (MAPK14), (**H**) liver protein kinase C alpha (PRKCα), and (**I**) liver inhibin subunit beta E (INHBE). NR: Basal diet without restricted IAA; R50: basal diet with 50% restricted IAA; R75: basal diet with 75% restricted IAA. The values are the means ± SE. *n* = 6–9 for NR, *n* = 5–7 for R50, and *n* = 7–8 for R75. ^#^ *p* ≤ 0.1 vs. NR. * *p* ≤ 0.05 vs. NR.

The mRNA abundance of hepatic FGF-21 and inhibin subunit beta E (INHBE) was significantly higher in R75 compared to NR (Figure 7A,I). Despite a numerical increase in protein expression of FGF-21 in the liver of R50 and R75, no significant differences were detected across groups (Figure 7B). In the liver, R50 and R75 had a greater transcript of activating transcription factor 4 (ATF4) and protein kinase C alpha (PRKCα) (Figure 7D,H), and R50 had a higher transcript of p38α mitogen-activated protein kinase (MAPK14) when compared to NR (Figure 7G). The gene expression of FGF-21 in the hypothalamus was higher for the R50 and R75 groups compared to NR (Figure 7C). Further, relative to NR, the R75 group had a greater expression of ATF4 and inositol-requiring enzyme type 1α (IRE1α) in the hypothalamus (Figure 7E,F). No significant differences were found in the gene expression of other molecules in the FGF-21 signaling pathway (Supplementary Figure S6).

## 4. Discussion

The greater concentration of dietary insulinogenic amino acids (IAA) is known to increase insulin secretion and hyperinsulinemia, which could eventually increase the risk of insulin resistance and T2D [21,22,23,24,25,26,27]. Little is known about whether the restriction of IAA could impact the glucose metabolism in peripheral tissues. The objective of this study was to examine the effect of IAA restriction on glucose metabolism in a piglet model. Our study revealed several important findings: (1) restriction of IAA by 75% (R75) improved glucose tolerance and, together with IAA restriction by 50% (R50), reduced blood insulin concentration and HOMA-IR, which is suggestive of improved insulin sensitivity following IAA restriction; (2) R75 increased thermal radiation, suggestive of increased energy expenditure, which could be due to AA imbalance or induced FGF-21 signaling and may contribute to improved insulin sensitivity under IAA restriction; (3) R75 decreased adipocytes number in white adipose tissue (WAT); (4) R75 increased the transcript of GLUT1, PFKL, PKLR in liver and GCK in WAT, indicating a higher uptake of glucose in liver and a greater glycolysis in both liver and WAT that may partly contribute to improved glucose tolerance under IAA restriction; (5) R75 increased the mRNA abundance of IRS1 and AKT1 in skeletal muscle, suggestive of enhanced insulin signaling that could explain the lower insulin concentration and improved insulin sensitivity during IAA restriction; (6) R75 increased the transcript of FAS in the liver and WAT, which is suggestive of an increased lipogenesis in the liver and WAT; (7) R75 increased the gene expression of FGF-21 in both the liver and hypothalamus and its upstream molecules, such as ATF4 and INHBE, which may contribute to increase energy expenditure and improved glucose tolerance during IAA restriction. Overall, our results indicate that IAA restriction improves glucose tolerance and insulin sensitivity, likely through several concurrent mechanisms, including enhanced energy expenditure, improved hepatic glycolysis and insulin signaling in skeletal muscle, and induced FGF-21 signaling in both the liver and hypothalamus.

Little is known about the effect of the restriction of a mixture of IAA on glucose tolerance and insulin sensitivity. Here, we showed that IAA restriction improved glucose tolerance and insulin sensitivity in neonatal pigs without having significant changes in body weight. This is consistent with previous studies showing that restriction of members of IAA, such as Leu, Ile, and Val, improves insulin sensitivity and glucose and lipid metabolism in murine models [27,29,30,31,32,33,34]. More specifically, both R75 and R50 groups reduced plasma insulin concentration and HOMA-IR. Likewise, previous studies have linked the elevated plasma IAA concentration to increased HOMA-IR values and impaired glucose tolerance in children, which is associated with diminished insulin sensitivity [97]. A mixture of Leu, Ile, Val, Lys, and Thr intake [22], as well as consumption of Phe [25] and Ala [21], has been linked to an increased risk of hyperinsulinemia. Unlike the R75, the pigs in R50 did not significantly improve the glucose tolerance following OGTT, which is suggestive of a sensing threshold for the level of AA restriction. We have previously reported similar differential responses to the levels of protein dilution in rats [96]. Improved glucose tolerance and insulin sensitivity following IAA restriction can be attributed to increased thermal radiation, improved glucose metabolism, and insulin and FGF-21 signaling in multiple tissues, as discussed in the following sections.

Limited data are available on the effect of AA restriction on energy expenditure in pigs. The R75 group increased thermal radiation at the end of the experiment and had a higher AUC for thermal radiation than NR. These results are aligned with previously published reports from our own lab and others on induced thermogenesis under AA restriction or protein dilution in pigs [54,55], birds [58,98], and rodents [27,96,99,100,101]. Increased thermal radiation in piglets fed with the R75 diet may be associated with AA imbalance and a greater transcript of hepatic and hypothalamic FGF-21 (see the sections below for the role of FGF-21 on induced thermogenesis under AA restriction). In our recent review, we highlighted several other mechanisms for induced thermogenesis under the deficiency of essential AA in mammals, including sympathetic flux to brown adipose tissue and serotonergic signaling [102]. Since brown adipose tissue is absent in pigs [103], further research is required to study the mechanisms of low AA-induced thermogenesis in pigs and, in particular, the role of FGF-21 in this phenomenon. Increased energy expenditure in response to IAA restriction may contribute to improved insulin sensitivity and decreased adipocyte number in white adipose tissue observed in pigs fed with R75, as low energy expenditure has been linked with increased insulin resistance and adiposity [104]. One may expect a lower body weight for the R75 group compared to the NR group due to having a higher thermal radiation; however, we failed to observe a difference in body weight between these two groups. The lack of difference in body weight between R75 and NR groups could be explained by a greater cumulative dry matter intake in R75 than in NR. A concurrent increase in energy intake and energy expenditure under protein deficiency and amino acids restriction has been well demonstrated in rodent models, as we previously reviewed [102]. Increased thermal radiation in the current study is believed to be correlated with increased energy expenditure; however, without direct energy expenditure measurement, there would be a limitation to the level of understanding we have on the possible mechanisms for IAA restriction-induced thermogenesis. Whether increased thermogenesis in response to IAA restriction is associated with upregulation of adrenergic receptors and UCP3 in skeletal muscle warrants further study. Measuring the fecal energy output and physical activity may further shed light on energy partitioning during IAA restriction.

The data on the effect of IAA restriction on glucose and lipid metabolism in peripheral tissues are scarce. Whether or not remodeling glucose and lipid metabolism contributes to improved insulin sensitivity under IAA restriction is poorly understood. R75 increased the transcript of GLUT1, PFKL, PKLR in the liver and GCK in WAT. Hepatic GLUT1 is an insulin-independent facilitated glucose transporter that is known to have a higher gene expression during diabetes and fasting state [105]. GLUT1 is the prominent hepatic glucose transporter in fetuses and early post-natal, and it is primarily involved in transporting glucose into cells [106,107]. Increased GLUT1 mRNA in the liver is suggestive of improved glucose uptake in pigs fed the R75 diet. Unlike GLUT1, hepatic GLUT4 mRNA tended to be lower in R75 compared to NR. GLUT4 is an insulin-dependent glucose transporter with a minor expression level in the liver [105], and its gene expression is likely decreased due to reduced circulating insulin concentrations in R75 pigs. GCK, PFKL, and PKLR are key genes involved in glycolysis. Greater expression of these genes under severe IAA restriction (R75) suggests an increased rate of glycolysis. In support of our data, previous studies have reported that increased consumption of BCAA is associated with impaired glucose metabolism [108]. Overall, increased glucose uptake and greater glycolysis in the liver and WAT may partly contribute to improved glucose tolerance and insulin sensitivity under IAA restriction.

In regard to lipid metabolism, R75 increased the transcript of FAS in the liver and WAT. FAS is a key gene in regulating lipid metabolism and is involved in the de novo synthesis of fatty acids. Higher FAS mRNA in liver and WAT is suggestive of an increased lipogenesis in these tissues when the R75 diet is offered. Likewise, previous research has shown that Lys restriction increases the rate of lipogenesis through the upregulation of several genes including sterol regulatory element binding protein-1c, fatty acid binding protein 4, and stearoyl CoA desaturase in bovine stromal vascular cells [109]. This is in line with our previous findings that low-protein diets increase the FAS mRNA in the liver of pigs and rats [60,96] and fat mass in birds [58]. This may be explained by an increased energy intake from carbohydrates and fats in an attempt to meet the protein needs in animals restricted to AA and proteins. Increased energy intake patterns are evident in the current study, with the R75 group having a higher cumulative dry matter intake than the NR group. Previous research supports the hyperphagic effects of low-protein and AA-restricted diets [102].

Although the effect of IAA restriction on insulin sensitivity has been previously documented, the insulin signaling at the cellular level has not been fully dissected. Here, we show that the IRS1 and AKT1 gene expression is upregulated in the skeletal muscle of pigs fed with the R75 diet. IRS1 and AKT play a key role in the insulin signaling pathway, and their downregulation is associated with insulin resistance [110,111]. Increased gene expression of IRS1 and AKT1 in the skeletal muscle of pigs fed with R75 is suggestive of enhanced insulin signaling in this group. In one study, prolonged exposure of skeletal muscle in vitro to high concentrations of BCAA leads to insulin resistance due to impaired IRS1/AKT signaling [112]. This is in agreement with our study that IRS1 and AKT1 mRNA are increased when IAA are restricted by 75%. Improved insulin signaling in the skeletal muscle of R75 pigs could explain the lower insulin concentration and improved insulin sensitivity during IAA restriction.

Previous studies have described several mechanisms for the beneficial effects of IAA on insulin sensitivity. Improvement in insulin sensitivity following BCAA restriction in humans and rodents has been linked with activated general control nonderepressible 2 (GCN2), AMP-activated protein kinase (AMPK), and AKT1 [29,44] and the decreased mammalian target of rapamycin (mTOR) and lipogenesis gene expression in adipose tissue [33,113,114]. Improved glucose tolerance and insulin sensitivity following IAA restriction in the piglet model may also be related to the upregulation of FGF-21. Improved glucose tolerance and insulin sensitivity following BCAA restriction have been related to increased blood concentration of FGF-21 in rodents [33,34,44,115]. To the best of our knowledge, this is the first study that has investigated the impact of IAA restriction on hepatic and hypothalamic FGF-21 signaling pathways in pigs. In this study, R75 pigs had a higher mRNA abundance of FGF-21 in both the liver and hypothalamus. FGF-21 is a member of the fibroblast growth factor (FGF) superfamily and is mainly expressed in the liver, which is known to play a key role in regulating glucose, lipid, and energy metabolism in multiple tissues, including the liver, heart, brain, adipose tissue, and skeletal muscle [116]. Administration of FGF-21 has been shown to improve hepatic insulin sensitivity and reduce blood glucose concentration in rodents [117,118]. Further, FGF-21 infusion results in increased energy expenditure and core body temperature in rodents [119,120,121]. In support of our findings, previous studies from our laboratory and others have shown that AA restriction [27,100,122], BCAA restriction [32,44,123], and protein dilution [96,99,124,125,126] increase either blood FGF-21 concentration or hepatic FGF-21 transcript in rodent models. Further, for the first time, we herein show that IAA restriction by 75% induces the expression of INHBE in the liver. Activin E is a secreted peptide encoded by the INHBE and is a member of the transforming growth factor beta (TGFβ) superfamily, which is mainly expressed in the liver [127,128,129,130]. Transgenic mice overexpressing activin E had a lower blood glucose level than control mice and showed a higher insulin sensitivity and improved glucose tolerance [131,132,133]. Activin E improves insulin sensitivity, increases energy expenditure, and protects from obesity [131,132,134,135,136,137]. Activin E likely enhances thermogenesis through stimulation of FGF-21 expression [131]. Greater expressions of hepatic FGF-21 and INHBE are likely to contribute to increased thermal radiation and improved glucose tolerance during IAA restriction.

Our results indicate that IAA restriction during early life improves glucose metabolism and insulin sensitivity in a neonatal piglet model. The data on the link between consumption of IAA in early life and the development of metabolic diseases in later stages in humans are scarce. Whether dietary IAA restriction improves metabolic health in human infants needs further investigation. In particular, that is of interest to study the effect of individual IAA restriction during neonatal periods on childhood obesity as well as changes in body composition and profile of metabolic biomarkers in blood in randomized controlled studies in individuals with overweight and obesity. As such, additional studies would be needed to investigate the long-term impact of IAA restriction on an individual’s health and risk of developing insulin resistance and other non-communicable diseases. In the current study, we demonstrated that the IAA restriction-induced effects on glucose tolerance and insulin sensitivity are associated with metabolic adaptations such as transcriptional remodeling of glucose metabolism, insulin signaling, and FGF-21 signaling in several tissues, including the liver, white adipose tissue, skeletal muscle, and hypothalamus. It has yet to be further studied whether similar adaptations occur when consumption of IAA is restricted in human neonates and whether these effects carry on until adolescence and even over the longer term.

## 5. Conclusions

To the best of our knowledge, this is the first study assessing the effect of dietary IAA restriction on glucose homeostasis and insulin sensitivity in a neonatal piglet model. We found that the restriction of IAA by 75% improved glucose tolerance and insulin sensitivity in piglets. Our findings suggest that this improvement is associated with improved glycolysis and glucose uptake in the liver, insulin signaling in skeletal muscle, and FGF-21 signaling in both the liver and hypothalamus. Further mechanistic studies are warranted to delineate the role of FGF-21 in IAA restriction-induced improvement in insulin sensitivity in pigs. There are still unanswered questions on the effect of IAA consumption on human infants and metabolic health in the long term. The molecular and cellular mechanisms underlying the IAA restriction-induced improvement in insulin sensitivity have not been fully studied in humans. Further research in this area may lead to the development of novel treatments reflecting the benefits of IAA restriction and can be potentially used to treat diabetes.

## Figures and Tables

**Table 1:** Dietary ingredients and calculated chemical composition (as-fed basis).

Ingredient % ^2^	Diets ^1^		
	NR	R50	R75
Whey protein concentrate 36.17%	6.30	6.30	6.30
Dried whey powder	10.00	10.00	10.00
Corn oil	14.80	14.80	14.80
Sodium casein AMCO	2.00	2.00	2.00
Dextrose	9.84	7.82	6.75
Lactose	28.39	28.39	28.39
Dicalcium phosphate 18.5%	3.50	3.50	3.50
L-arginine	0.60	0.23	0.04
L-alanine	0.90	0.31	0.05
L-glutamic acid	14.05	21.68	25.5
L-histidine	0.37	0.37	0.37
L-isoleucine	0.89	0.30	0.01
L-leucine	1.83	0.68	0.11
L-lysine HCL	1.83	0.69	0.13
DL-methionine	0.43	0.43	0.43
L-phenylalanine	0.87	0.34	0.08
L-threonine	0.92	0.33	0.04
L-tryptophan	0.26	0.26	0.26
L-valine	1.01	0.36	0.03
Vitamin premix	0.13	0.13	0.13
Mineral premix	0.18	0.18	0.18
Salt	0.90	0.90	0.90
**Calculated Chemical Composition**			
Dry matter, %	96.65	96.88	97.00
ME ^3^, Mcal/kg	4.10	4.10	4.10
Crude protein, %	22.00	22.00	22.00
Crude fat, %	15.21	15.21	15.21
Calcium, %	0.98	0.98	0.98
Total phosphorus, %	0.75	0.75	0.75
Potassium, %	0.29	0.29	0.29
SID ^4^ alanine, %	1.02	0.51	0.25
SID ^4^ arginine, %	0.74	0.37	0.18
SID ^4^ glutamic acid, %	14.92	22.56	26.37
SID ^4^ histidine, %	0.48	0.48	0.48
SID ^4^ isoleucine, %	1.18	0.59	0.30
SID ^4^ leucine, %	2.30	1.15	0.58
SID ^4^ lysine, %	2.10	1.05	0.53
SID ^4^ methionine, %	0.54	0.54	0.54
SID ^4^ phenylalanine, %	1.05	0.52	0.26
SID ^4^ threonine, %	1.18	0.59	0.30
SID ^4^ tryptophan, %	0.34	0.34	0.34
SID ^4^ valine, %	1.31	0.66	0.33

^1^ NR: basal diet without restricted insulinogenic amino acid (IAA); R50: basal diet with 50% restricted IAA; R75: basal diet with 75% restricted IAA. ^2^ Whey protein concentrate, dried whey, corn oil, salt, dextrose, dicalcium phosphate, lactose, and L-arginine, DL-Methionine (99%), L-Lysine HCl and vitamin and mineral premix were obtained from Nutra Blend, LLC (Neosho, MO, USA). Sodium caseinate was obtained from AMCO PROTEIN (Burlington, NJ, USA). L-Lysine, L-Tryptophan (98%), and L-threonine (98.5%) were obtained from Ajinomoto (Overland Park, KS, USA). L-valine (96.5), L-histidine, L-phenylalanine, L-isoleucine (98.5), L-glutamic acid, L-leucine, and L-alanine were purchased from Ajinomoto Health & Nutrition North America, Inc. (Raleigh, NC, USA). Vitamin premix contained (per kg): vitamin A, 1,653,750 IU; vitamin D_3_, 661,500 IU; vitamin E, 17,640 IU; vitamin K, 1323 mg; vitamin B12, 13.23 mg; niacin, 19,845 mg; pantothenic acid, 11,025 mg; riboflavin, 3307.5 mg; phytase, 300,056.4 FYT. Mineral premix contained: copper, 11,000 ppm; iodine, 198 ppm; iron, 73,000 ppm; manganese, 22,000 ppm; selenium, 198 ppm; zinc, 73,000 ppm. ^3^ ME: Metabolizable Energy. ^4^ SID: Standard Ileal Digestibility.

**Table 2:** Analyzed chemical composition of diets (dry weight basis).

Items	Diets ^1^		
	NR	R50	R75
Arginine, %	0.81	0.35	0.19
Alanine, %	0.71	0.30	0.24
Aspartic acid, %	0.62	0.51	0.59
Cysteine, %	0.11	0.10	0.10
Glutamic acid, %	16.28	22.69	25.06
Glycine, %	0.14	0.11	0.11
Histidine, %	0.59	0.43	0.36
Hydroxylysine, %	0.00	0.02	0.06
Hydroxyproline, %	0.00	0.02	0.00
Isoleucine, %	1.36	0.58	0.38
Lanthionine ^2^, %	0.18	0.21	0.07
Leucine, %	1.80	1.21	0.53
Lysine, %	1.07	0.58	0.45
Methionine, %	0.66	0.43	0.44
Ornithine ^2^, %	0.01	0.00	0.00
Phenylalanine, %	0.86	0.34	0.29
Proline, %	0.49	0.32	0.36
Serine, %	0.31	0.22	0.23
Taurine ^2^, %	0.16	0.16	0.14
Threonine, %	1.20	0.55	0.31
Tryptophan, %	0.33	0.35	0.32
Tyrosine, %	0.28	0.20	0.22
Valine, %	1.35	0.65	0.34
Dry matter, %	95.89	96.78	97.36
Crude protein ^3^, %	22.32	22.29	22.55
Calcium, %	0.91	0.90	0.98
Phosphorus, %	0.85	0.77	0.83

^1^ NR: basal diet without restricted insulinogenic amino acid (IAA); R50: basal diet with 50% restricted IAA; R75: basal diet with 75% restricted IAA. ^2^ Non-proteinogenic amino acid. ^3^ Crude protein = %N × 6.25.

## Data Availability

The original contributions presented in this study are included in the article/supplementary material. Further inquiries can be directed to the corresponding author.

## Supplementary Materials

The following supporting information can be downloaded at: https://www.mdpi.com/article/10.3390/nu17101675/s1, Table S1. The Genes, sequences forward (F) and reverse (R), amplicon size (bp), location on template, and GenBank accession numbers for primers used for reverse transcription qualitative real-time polymerase chain reaction (RT-qPCR); Table S2. Primary and secondary antibodies for immunoblotting, host, dilution, and supplier; Table S3. Effect of insulinogenic amino acid restriction on dry matter intake and growth parameters of neonatal pigs; Figure S1. Effect of insulinogenic amino acids restriction (IAA) on glucose tolerance; Figure S2. Effect of insulinogenic amino acids (IAA) restriction on mRNA abundance of genes involved in glucose transport and metabolism in the liver, white adipose tissue (WAT), and skeletal muscle of neonatal pigs; Figure S3. Effect of insulinogenic amino acids (IAA) restriction on mRNA abundance of genes involved in glycogen metabolism in the liver and skeletal muscle of neonatal pigs; Figure S4. Effect of insulinogenic amino acids (IAA) restriction on mRNA abundance of genes involved in insulin signaling in the liver, white adipose tissue (WAT), and skeletal muscle of neonatal pigs; Figure S5. Effect of insulinogenic amino acids (IAA) restriction on mRNA abundance of genes involved in lipid metabolism in the liver, white adipose tissue (WAT), and skeletal muscle of neonatal pigs; Figure S6. Effect of insulinogenic amino acids (IAA) restriction on mRNA abundance of genes involved in FGF-21 pathway in the liver, white adipose tissue (WAT), and hypothalamus of neonatal pigs.

## Abbreviations

The abbreviations below were used in this manuscript.

AA	Amino Acids
ADG	Average Daily Gain
ADMI	Average Dry Matter Intake
CDMI	Cumulative Dry Matter Intake
ADPI	Average Daily Protein Intake
Ala	Alanine
Arg	Arginine
BCAA	Branched Chain Amino Acids
DM	Dry Matter
G:F	Gain-to-Feed Ratio
G:P	Gain-to-Protein Ratio
HOMA-IR	Homeostatic Model Assessment for Insulin Resistance
IAA	Insulinogenic Amino Acids
Ile	Isoleucine
Leu	Leucine
Lys	lysine
NR	No Restriction
OGTT	Oral Glucose Tolerance Test
Phe	Phenylalanine
qPCR	Real-Time Quantitative PCR
QUICKI	Quantitative Insulin Sensitivity Check Index
T2D	Type 2 Diabetes
Thr	Threonine
Val	Valine

## References

[1] NCD Risk Factor Collaboration (NCD-RisC) (2017). Worldwide Trends in Body-Mass Index, Underweight, Overweight, and Obesity from 1975 to 2016: A Pooled Analysis of 2416 Population-Based Measurement Studies in 128·9 Million Children, Adolescents, and Adults. Lancet.

[2] de Onis M., Blössner M., Borghi E. (2010). Global Prevalence and Trends of Overweight and Obesity among Preschool Children1234. Am. J. Clin. Nutr..

[3] Mayer-Davis E.J., Lawrence J.M., Dabelea D., Divers J., Isom S., Dolan L., Imperatore G., Linder B., Marcovina S., Pettitt D.J. (2017). Incidence Trends of Type 1 and Type 2 Diabetes among Youths, 2002–2012. N. Engl. J. Med..

[4] Dabelea D., Mayer-Davis E.J., Saydah S., Imperatore G., Linder B., Divers J., Bell R., Badaru A., Talton J.W., Crume T. (2014). Prevalence of Type 1 and Type 2 Diabetes among Children and Adolescents from 2001 to 2009. JAMA.

[5] Sanyaolu A., Okorie C., Qi X., Locke J., Rehman S. (2019). Childhood and Adolescent Obesity in the United States: A Public Health Concern. Glob. Pediatr. Health.

[6] Andes L.J., Cheng Y.J., Rolka D.B., Gregg E.W., Imperatore G. (2020). Prevalence of Prediabetes Among Adolescents and Young Adults in the United States, 2005–2016. JAMA Pediatr..

[7] Divers J., Mayer-Davis E.J., Lawrence J.M., Isom S., Dabelea D., Dolan L., Imperatore G., Marcovina S., Pettitt D.J., Pihoker C. (2020). Trends in Incidence of Type 1 and Type 2 Diabetes Among Youths-Selected Counties and Indian Reservations, United States, 2002–2015. MMWR Morb. Mortal. Wkly. Rep..

[8] Centers for Disease Control (2020). National Diabetes Statistics Report 2020: Estimates of Diabetes and Its Burden in the United States. https://stacks.cdc.gov/view/cdc/85309.

[9] Imperatore G., Boyle J.P., Thompson T.J., Case D., Dabelea D., Hamman R.F., Lawrence J.M., Liese A.D., Liu L.L., Mayer-Davis E.J. (2012). Projections of Type 1 and Type 2 Diabetes Burden in the U.S. Population Aged <20 Years through 2050: Dynamic Modeling of Incidence, Mortality, and Population Growth. Diabetes Care.

[10] Rolland-Cachera M.-F., Deheeger M., Akrout M., Bellisle F. (1995). Influence of Macronutrients on Adiposity Development: A Follow up Study of Nutrition and Growth from 10 Months to 8 Years of Age. Int. J. Obes. Relat. Metab. Disord..

[11] Koletzko B., von Kries R., Closa R., Escribano J., Scaglioni S., Giovannini M., Beyer J., Demmelmair H., Gruszfeld D., Dobrzanska A. (2009). Lower Protein in Infant Formula Is Associated with Lower Weight up to Age 2 y: A Randomized Clinical Trial. Am. J. Clin. Nutr..

[12] Koletzko B., Von Kries R., Monasterolo R.C., Subías J.E., Scaglioni S., Giovannini M., Beyer J., Demmelmair H., Anton B., Gruszfeld D. (2009). Can Infant Feeding Choices Modulate Later Obesity Risk?. Am. J. Clin. Nutr..

[13] Brands B., Demmelmair H., Koletzko B., EarlyNutrition P. (2014). How Growth Due to Infant Nutrition Influences Obesity and Later Disease Risk. Acta Paediatr..

[14] Michaelsen K.F., Greer F.R. (2014). Protein Needs Early in Life and Long-Term Health. Am. J. Clin. Nutr..

[15] Camier A., Davisse-Paturet C., Scherdel P., Lioret S., Heude B., Charles M.A., de Lauzon-Guillain B. (2021). Early Growth According to Protein Content of Infant Formula: Results from the EDEN and ELFE Birth Cohorts. Pediatr. Obes..

[16] Weber M., Grote V., Closa-Monasterolo R., Escribano J., Langhendries J.P., Dain E., Giovannini M., Verduci E., Gruszfeld D., Socha P. (2014). Lower Protein Content in Infant Formula Reduces BMI and Obesity Risk at School Age: Follow-up of a Randomized Trial. Am. J. Clin. Nutr..

[17] Kouwenhoven S.M.P., Antl N., Finken M.J.J., Twisk J.W.R., van der Beek E.M., Abrahamse-Berkeveld M., van de Heijning B.J.M., van Goudoever J.B., Koletzko B.V. (2021). Long-Term Effects of a Modified, Low-Protein Infant Formula on Growth and Body Composition: Follow-up of a Randomized, Double-Blind, Equivalence Trial. Clin. Nutr..

[18] Kouwenhoven S.M.P., Muts J., Finken M.J.J., van Goudoever J.B. (2022). Low-Protein Infant Formula and Obesity Risk. Nutrients.

[19] Koletzko B., Demmelmair H., Grote V., Prell C., Weber M. (2016). High Protein Intake in Young Children and Increased Weight Gain and Obesity Risk. Am. J. Clin. Nutr..

[20] Kalhan S.C. (2009). Optimal Protein Intake in Healthy Infants. Am. J. Clin. Nutr..

[21] Newsholme P., Brennan L., Bender K. (2006). Amino Acid Metabolism, β-Cell Function, and Diabetes. Diabetes.

[22] Nilsson M., Holst J.J., Björck I.M. (2007). Metabolic Effects of Amino Acid Mixtures and Whey Protein in Healthy Subjects: Studies Using Glucose-Equivalent Drinks2. Am. J. Clin. Nutr..

[23] Kalogeropoulou D., LaFave L., Schweim K., Gannon M.C., Nuttall F.Q. (2009). Lysine Ingestion Markedly Attenuates the Glucose Response to Ingested Glucose without a Change in Insulin Response. Am. J. Clin. Nutr..

[24] van Sloun B., Goossens G.H., Erdos B., Lenz M., van Riel N., Arts I.C.W. (2020). The Impact of Amino Acids on Postprandial Glucose and Insulin Kinetics in Humans: A Quantitative Overview. Nutrients.

[25] Zhou Q., Sun W.-W., Chen J.-C., Zhang H.-L., Liu J., Lin Y., Lin P.-C., Wu B.-X., An Y.-P., Huang L. (2022). Phenylalanine Impairs Insulin Signaling and Inhibits Glucose Uptake through Modification of IRβ. Nat. Commun..

[26] Anthony T.G., Morrison C.D., Gettys T.W. (2013). Remodeling of Lipid Metabolism by Dietary Restriction of Essential Amino Acids. Diabetes.

[27] Yap Y.W., Rusu P.M., Chan A.Y., Fam B.C., Jungmann A., Solon-Biet S.M., Barlow C.K., Creek D.J., Huang C., Schittenhelm R.B. (2020). Restriction of Essential Amino Acids Dictates the Systemic Metabolic Response to Dietary Protein Dilution. Nat. Commun..

[28] Kuhara T., Ikeda S., Ohneda A., Sasaki Y. (1991). Effects of Intravenous Infusion of 17 Amino Acids on the Secretion of GH, Glucagon, and Insulin in Sheep. Am. J. Physiol..

[29] Xiao F., Huang Z., Li H., Yu J., Wang C., Chen S., Meng Q., Cheng Y., Gao X., Li J. (2011). Leucine Deprivation Increases Hepatic Insulin Sensitivity via GCN2/mTOR/S6K1 and AMPK Pathways. Diabetes.

[30] Xiao F., Yu J., Guo Y., Deng J., Li K., Du Y., Chen S., Zhu J., Sheng H., Guo F. (2014). Effects of Individual Branched-Chain Amino Acids Deprivation on Insulin Sensitivity and Glucose Metabolism in Mice. Metabolism.

[31] Fontana L., Cummings N.E., Arriola Apelo S.I., Neuman J.C., Kasza I., Schmidt B.A., Cava E., Spelta F., Tosti V., Syed F.A. (2016). Decreased Consumption of Branched-Chain Amino Acids Improves Metabolic Health. Cell. Rep..

[32] Cummings N.E., Williams E.M., Kasza I., Konon E.N., Schaid M.D., Schmidt B.A., Poudel C., Sherman D.S., Yu D., Arriola Apelo S.I. (2018). Restoration of Metabolic Health by Decreased Consumption of Branched-Chain Amino Acids. J. Physiol..

[33] Karusheva Y., Koessler T., Strassburger K., Markgraf D., Mastrototaro L., Jelenik T., Simon M.C., Pesta D., Zaharia O.P., Bodis K. (2019). Short-Term Dietary Reduction of Branched-Chain Amino Acids Reduces Meal-Induced Insulin Secretion and Modifies Microbiome Composition in Type 2 Diabetes: A Randomized Controlled Crossover Trial. Am. J. Clin. Nutr..

[34] Yu D., Richardson N.E., Green C.L., Spicer A.B., Murphy M.E., Flores V., Jang C., Kasza I., Nikodemova M., Wakai M.H. (2021). The Adverse Metabolic Effects of Branched-Chain Amino Acids Are Mediated by Isoleucine and Valine. Cell. Metab..

[35] Miller E.R., Ullrey D.E. (1987). The Pig as a Model for Human Nutrition. Annu. Rev. Nutr..

[36] Puiman P., Stoll B. (2008). Animal Models to Study Neonatal Nutrition in Humans. Curr. Opin. Clin. Nutr. Metab. Care.

[37] Bellinger D.A., Merricks E.P., Nichols T.C. (2006). Swine Models of Type 2 Diabetes Mellitus: Insulin Resistance, Glucose Tolerance, and Cardiovascular Complications. ILAR J..

[38] Harwood H.J., Listrani P., Wagner J.D. (2012). Nonhuman Primates and Other Animal Models in Diabetes Research. J. Diabetes Sci. Technol..

[39] McGlone J. (2010). Guide for the Care and Use of Agricultural Animals in Research and Teaching.

[40] Rafiee-Tari N., Fan M.Z., Archbold T., Arranz E., Corredig M. (2019). Effect of Milk Protein Composition and Amount of Beta-Casein on Growth Performance, Gut Hormones, and Inflammatory Cytokines in an in Vivo Piglet Model. J. Dairy Sci..

[41] Goodarzi P., Habibi M., Roberts K., Sutton J., Shili C.N., Lin D., Pezeshki A. (2021). Dietary Tryptophan Supplementation Alters Fat and Glucose Metabolism in a Low-Birthweight Piglet Model. Nutrients.

[42] Calbet J.A.L. (2002). Plasma Glucagon and Insulin Responses Depend on the Rate of Appearance of Amino Acids after Ingestion of Different Protein Solutions in Humans. J. Nutr..

[43] Nilsson M. (2004). Glycemia and Insulinemia in Healthy Subjects after Lactose-Equivalent Meals of Milk and Other Food Proteins: The Role of Plasma Amino Acids and Incretins. Am. J. Clin. Nutr..

[44] Wanders D., Stone K.P., Dille K., Simon J., Pierse A., Gettys T.W. (2015). Metabolic Responses to Dietary Leucine Restriction Involve Remodeling of Adipose Tissue and Enhanced Hepatic Insulin Signaling. Biofactors.

[45] Lee B.C., Kaya A., Ma S., Kim G., Gerashchenko M.V., Yim S.H., Hu Z., Harshman L.G., Gladyshev V.N. (2014). Methionine Restriction Extends Lifespan of Drosophila Melanogaster under Conditions of Low Amino-Acid Status. Nat. Commun..

[46] Lees E.K., Banks R., Cook C., Hill S., Morrice N., Grant L., Mody N., Delibegovic M. (2017). Direct Comparison of Methionine Restriction with Leucine Restriction on the Metabolic Health of C57BL/6J Mice. Sci. Rep..

[47] Zhou Z., Yin H., Guo Y., Fang Y., Yuan F., Chen S., Guo F. (2021). A Fifty Percent Leucine-Restricted Diet Reduces Fat Mass and Improves Glucose Regulation. Nutr. Metab..

[48] Ebert A.R., Berman A.S., Harrell R.J., Kessler A.M., Cornelius S.G., Odle J. (2005). Vegetable Proteins Enhance the Growth of Milk-Fed Piglets, despite Lower Apparent Ileal Digestibility. J. Nutr..

[49] DeRouchey J.M., Goodband R.D., Tokach M.D., Nelssen J.L., Dritz S.S., Meisinger D.J. (2010). Nursery Swine Nutrient Recommendations and Feeding Management. National Swine Nutrition Guide.

[50] Kim S.W., Wu G. (2004). Dietary Arginine Supplementation Enhances the Growth of Milk-Fed Young Pigs. J. Nutr..

[51] Madsen J.G., Mueller S., Kreuzer M., Bigler M.B., Silacci P., Bee G. (2018). Milk Replacers Supplemented with Either L-Arginine or L-Carnitine Potentially Improve Muscle Maturation of Early Reared Low Birth Weight Piglets from Hyperprolific Sows. Animal.

[52] Montelius C., Szwiec K., Kardas M., Lozinska L., Erlanson-Albertsson C., Pierzynowski S., Rehfeld J.F., Weström B. (2014). Dietary Thylakoids Suppress Blood Glucose and Modulate Appetite-Regulating Hormones in Pigs Exposed to Oral Glucose Tolerance Test. Clin. Nutr..

[53] Habibi M., Shili C., Sutton J., Goodarzi P., Maylem E.R., Spicer L., Pezeshki A. (2021). Branched-Chain Amino Acids Partially Recover the Reduced Growth of Pigs Fed with Protein-Restricted Diets through Both Central and Peripheral Factors. Anim. Nutr..

[54] Spring S., Premathilake H., Bradway C., Shili C., Desilva U., Carter S., Pezeshki A. (2020). Effect of Very Low-Protein Diets Supplemented with Branched-Chain Amino Acids on Energy Balance, Plasma Metabolomics and Fecal Microbiome of Pigs. Sci. Rep..

[55] Spring S., Premathilake H., Desilva U., Shili C., Carter S., Pezeshki A. (2020). Low Protein-High Carbohydrate Diets Alter Energy Balance, Gut Microbiota Composition and Blood Metabolomics Profile in Young Pigs. Sci. Rep..

[56] Shili C.N., Broomhead J.N., Spring S.C., Lanahan M.B., Pezeshki A. (2020). A Novel Corn-Expressed Phytase Improves Daily Weight Gain, Protein Efficiency Ratio and Nutrients Digestibility and Alters Fecal Microbiota in Pigs Fed with Very Low Protein Diets. Animals.

[57] Goodarzi P., Wileman C.M., Habibi M., Walsh K., Sutton J., Shili C.N., Chai J., Zhao J., Pezeshki A. (2022). Effect of Isoleucine and Added Valine on Performance, Nutrients Digestibility and Gut Microbiota Composition of Pigs Fed with Very Low Protein Diets. Int. J. Mol. Sci..

[58] Sutton J., Habibi M., Shili C.N., Beker A., Salak-Johnson J.L., Foote A., Pezeshki A. (2024). Low-Protein Diets Differentially Regulate Energy Balance during Thermoneutral and Heat Stress in Cobb Broiler Chicken (Gallus Domesticus). Int. J. Mol. Sci..

[59] Habibi M., Goodarzi P., Shili C.N., Sutton J., Wileman C.M., Kim D.M., Lin D., Pezeshki A. (2022). A Mixture of Valine and Isoleucine Restores the Growth of Protein-Restricted Pigs Likely through Improved Gut Development, Hepatic IGF-1 Pathway, and Plasma Metabolomic Profile. Int. J. Mol. Sci..

[60] Goodarzi P., Habibi M., Gorton M.W., Walsh K., Tarkesh F., Fuhrig M., Pezeshki A. (2023). Dietary Isoleucine and Valine: Effects on Lipid Metabolism and Ureagenesis in Pigs Fed with Protein Restricted Diets. Metabolites.

[61] Matthews D.R., Hosker J.P., Rudenski A.S., Naylor B.A., Treacher D.F., Turner R.C. (1985). Homeostasis Model Assessment: Insulin Resistance and β-Cell Function from Fasting Plasma Glucose and Insulin Concentrations in Man. Diabetologia.

[62] Katz A., Nambi S.S., Mather K., Baron A.D., Follmann D.A., Sullivan G., Quon M.J. (2000). Quantitative Insulin Sensitivity Check Index: A Simple, Accurate Method for Assessing Insulin Sensitivity In Humans. J. Clin. Endocr. Metab..

[63] Parlee S.D., Lentz S.I., Mori H., MacDougald O.A. (2014). Quantifying Size and Number of Adipocytes in Adipose Tissue. Methods Enzymol..

[64] Shili C.N., Habibi M., Sutton J., Barnes J., Burch-Konda J., Pezeshki A. (2021). Effect of a Phytogenic Water Additive on Growth Performance, Blood Metabolites and Gene Expression of Amino Acid Transporters in Nursery Pigs Fed with Low-Protein/High-Carbohydrate Diets. Animals.

[65] Pezeshki A., Muench G.P., Chelikani P.K. (2012). Short Communication: Expression of Peptide YY, Proglucagon, Neuropeptide Y Receptor Y2, and Glucagon-like Peptide-1 Receptor in Bovine Peripheral Tissues. J. Dairy Sci..

[66] Habibi M., Shili C.N., Sutton J., Goodarzi P., Pezeshki A. (2022). Dietary Branched-Chain Amino Acids Modulate the Dynamics of Calcium Absorption and Reabsorption in Protein-Restricted Pigs. J. Anim. Sci. Biotechnol..

[67] Vitali M., Dimauro C., Sirri R., Zappaterra M., Zambonelli P., Manca E., Sami D., Lo Fiego D.P., Davoli R. (2018). Effect of Dietary Polyunsaturated Fatty Acid and Antioxidant Supplementation on the Transcriptional Level of Genes Involved in Lipid and Energy Metabolism in Swine. PLoS ONE.

[68] Kim S.-C., Jang H.-C., Lee S.-D., Jung H.-J., Park J.-C., Lee S.-H., Kim T.-H., Choi B.-H. (2014). Changes in Expression of Insulin Signaling Pathway Genes by Dietary Fat Source in Growing-Finishing Pigs. J. Anim. Sci. Technol..

[69] Zhao L., Guo H., Sun H. (2020). Effects of Low-Protein Diet Supplementation with Alpha-Ketoglutarate on Growth Performance, Nitrogen Metabolism and mTOR Signalling Pathway of Skeletal Muscle in Piglets. J. Anim. Physiol. Anim. Nutr..

[70] Chomwisarutkun K., Murani E., Ponsuksili S., Wimmers K. (2012). Gene Expression Analysis of Mammary Tissue during Fetal Bud Formation and Growth in Two Pig Breeds--Indications of Prenatal Initiation of Postnatal Phenotypic Differences. BMC. Dev. Biol..

[71] Xie C., Wang Q., Wang J., Tan B., Fan Z., Deng Z., Wu X., Yin Y. (2016). Developmental Changes in Hepatic Glucose Metabolism in a Newborn Piglet Model: A Comparative Analysis for Suckling Period and Early Weaning Period. Biochem. Biophys. Res. Commun..

[72] Cervantes M., Cota M., Arce N., Castillo G., Avelar E., Espinoza S., Morales A. (2016). Effect of Heat Stress on Performance and Expression of Selected Amino Acid and Glucose Transporters, HSP90, Leptin and Ghrelin in Growing Pigs. J. Therm. Biol..

[73] Fang L., Jiang X., Su Y., Zhu W. (2014). Long-Term Intake of Raw Potato Starch Decreases Back Fat Thickness and Dressing Percentage but Has No Effect on the Longissimus Muscle Quality of Growing–Finishing Pigs. Livest. Sci..

[74] Espinosa C.D., Fry R.S., Kocher M.E., Stein H.H. (2020). Effects of Copper Hydroxychloride on Growth Performance and Abundance of Genes Involved in Lipid Metabolism of Growing Pigs. J. Anim. Sci..

[75] Gavalda-Navarro A., Pastor J.J., Mereu A., Villarroya F., Ipharraguerre I.R. (2018). Developmental Regulation of the Intestinal FGF19 System in Domestic Pigs. Am. J. Physiol. Gastrointest. Liver Physiol..

[76] Merz T., Denoix N., Wigger D., Waller C., Wepler M., Vettorazzi S., Tuckermann J., Radermacher P., McCook O. (2020). The Role of Glucocorticoid Receptor and Oxytocin Receptor in the Septic Heart in a Clinically Relevant, Resuscitated Porcine Model With Underlying Atherosclerosis. Front. Endocrinol..

[77] Weber T.E., Kerr B.J., Spurlock M.E. (2008). Regulation of Hepatic Peroxisome Proliferator-Activated Receptor Alpha Expression but Not Adiponectin by Dietary Protein in Finishing Pigs. J. Anim. Physiol. Anim. Nutr..

[78] Jin J.-X., Lee S., Taweechaipaisankul A., Kim G.A., Lee B.C. (2017). Melatonin Regulates Lipid Metabolism in Porcine Oocytes. J. Pineal Res..

[79] Niu Y.-J., Zhou W., Nie Z.-W., Shin K.-T., Cui X.-S. (2020). Melatonin Enhances Mitochondrial Biogenesis and Protects against Rotenone-Induced Mitochondrial Deficiency in Early Porcine Embryos. J. Pineal Res..

[80] Duran-Montgé P., Theil P.K., Lauridsen C., Esteve-Garcia E. (2009). Dietary Fat Source Affects Metabolism of Fatty Acids in Pigs as Evaluated by Altered Expression of Lipogenic Genes in Liver and Adipose Tissues. Animal.

[81] Chen G., Zhang J., Zhang Y., Liao P., Li T., Chen L., Yin Y., Wang J., Wu G. (2014). Oral MSG Administration Alters Hepatic Expression of Genes for Lipid and Nitrogen Metabolism in Suckling Piglets. Amino Acids.

[82] Zhou X., Wan D., Zhang Y., Zhang Y., Long C., Chen S., He L., Tan B., Wu X., Yin Y. (2017). Diurnal Variations in Polyunsaturated Fatty Acid Contents and Expression of Genes Involved in Their de Novo Synthesis in Pigs. Biochem. Biophys. Res. Commun..

[83] He D., Ma J., Long K., Wang X., Li X., Jiang A., Li M. (2017). Differential Expression of Genes Related to Glucose Metabolism in Domesticated Pigs and Wild Boar. Biosci. Biotechnol. Biochem..

[84] Hu H., Li Y., Yang Y., Xu K., Yang L., Qiao S., Pan H. (2022). Effect of a Plateau Environment on the Oxidation State of the Heart and Liver through AMPK/P38 MAPK/Nrf2-ARE Signaling Pathways in Tibetan and DLY Pigs. Animals.

[85] Hu Y., Xu J., Shi S.J., Zhou X., Wang L., Huang L., Gao L., Pang W., Yang G., Chu G. (2022). Fibroblast Growth Factor 21 (FGF21) Promotes Porcine Granulosa Cell Estradiol Production and Proliferation via PI3K/AKT/mTOR Signaling. Theriogenology.

[86] He L., Kim T., Long Q., Liu J., Wang P., Zhou Y., Ding Y., Prasain J., Wood P.A., Yang Q. (2012). Carnitine Palmitoyltransferase-1b Deficiency Aggravates Pressure Overload-Induced Cardiac Hypertrophy Caused by Lipotoxicity. Circulation.

[87] Ying Z., Zhang H., Su W., Zhou L., Wang F., Li Y., Zhang L., Wang T. (2017). Dietary Methionine Restriction Alleviates Hyperglycemia in Pigs with Intrauterine Growth Restriction by Enhancing Hepatic Protein Kinase B Signaling and Glycogen Synthesis. J. Nutr..

[88] Li S., Wang H., Wang X., Wang Y., Feng J. (2017). Betaine Affects Muscle Lipid Metabolism via Regulating the Fatty Acid Uptake and Oxidation in Finishing Pig. J. Anim. Sci. Biotechnol..

[89] Zorrilla L.M., Irvin M.S., Gadsby J.E. (2009). Protein Kinase C Isoforms in the Porcine Corpus Luteum: Temporal and Spatial Expression Patterns. Domest. Anim. Endocrinol..

[90] Yin J., Liu M., Ren W., Duan J., Yang G., Zhao Y., Fang R., Chen L., Li T., Yin Y. (2015). Effects of Dietary Supplementation with Glutamate and Aspartate on Diquat-Induced Oxidative Stress in Piglets. PLoS ONE.

[91] Zhou H., Yu B., Gao J., Htoo J.K., Chen D. (2018). Regulation of Intestinal Health by Branched-Chain Amino Acids. Anim. Sci. J..

[92] Zhong H., Fan S., Du Y., Zhang Y., Zhang A., Jiang D., Han S., Wan B., Zhang G. (2022). African Swine Fever Virus MGF110-7L Induces Host Cell Translation Suppression and Stress Granule Formation by Activating the PERK/PKR-eIF2α Pathway. Microbiol. Spectr..

[93] Zheng Y., Zhang B., Guan H., Jiao X., Yang J., Cai J., Liu Q., Zhang Z. (2021). Selenium Deficiency Causes Apoptosis through Endoplasmic Reticulum Stress in Swine Small Intestine. Biofactors.

[94] Guo M., Wu M.H., Korompai F., Yuan S.Y. (2003). Upregulation of PKC Genes and Isozymes in Cardiovascular Tissues during Early Stages of Experimental Diabetes. Physiol. Genom..

[95] Pezeshki A., Chelikani P.K. (2014). Effects of Roux-En-Y Gastric Bypass and Ileal Transposition Surgeries on Glucose and Lipid Metabolism in Skeletal Muscle and Liver. Surg. Obes. Relat. Dis..

[96] Pezeshki A., Zapata R.C., Singh A., Yee N.J., Chelikani P.K. (2016). Low Protein Diets Produce Divergent Effects on Energy Balance. Sci. Rep..

[97] Suzuki Y., Kido J., Matsumoto S., Shimizu K., Nakamura K. (2019). Associations among Amino Acid, Lipid, and Glucose Metabolic Profiles in Childhood Obesity. BMC Pediatr..

[98] Buyse J., Decuypere E., Berghman L., Kühn E.R., Vandesande F. (1992). Effect of Dietary Protein Content on Episodic Growth Hormone Secretion and on Heat Production of Male Broiler Chickens. Br. Poult. Sci..

[99] Zapata R.C., Singh A., Pezeshki A., Avirineni B.S., Patra S., Chelikani P.K. (2019). Low-Protein Diets with Fixed Carbohydrate Content Promote Hyperphagia and Sympathetically Mediated Increase in Energy Expenditure. Mol. Nutr. Food Res..

[100] Zapata R.C., Singh A., Pezeshki A., Chelikani P.K. (2019). Tryptophan Restriction Partially Recapitulates the Age-Dependent Effects of Total Amino Acid Restriction on Energy Balance in Diet-Induced Obese Rats. J. Nutr. Biochem..

[101] Spring S., Singh A., Zapata R.C., Chelikani P.K., Pezeshki A. (2019). Methionine Restriction Partly Recapitulates the Sympathetically Mediated Enhanced Energy Expenditure Induced by Total Amino Acid Restriction in Rats. Nutrients.

[102] Pezeshki A., Chelikani P.K. (2021). Low Protein Diets and Energy Balance: Mechanisms of Action on Energy Intake and Expenditure. Front. Nutr..

[103] Trayhurn P., Temple N.J., Van Aerde J. (1989). Evidence from Immunoblotting Studies on Uncoupling Protein That Brown Adipose Tissue Is Not Present in the Domestic Pig. Can. J. Physiol. Pharmacol..

[104] Klaman L.D., Boss O., Peroni O.D., Kim J.K., Martino J.L., Zabolotny J.M., Moghal N., Lubkin M., Kim Y.B., Sharpe A.H. (2000). Increased Energy Expenditure, Decreased Adiposity, and Tissue-Specific Insulin Sensitivity in Protein-Tyrosine Phosphatase 1B-Deficient Mice. Mol. Cell. Biol..

[105] Chadt A., Al-Hasani H. (2020). Glucose Transporters in Adipose Tissue, Liver, and Skeletal Muscle in Metabolic Health and Disease. Pflugers Arch.-Eur. J. Physiol..

[106] Gould G.W. (1997). Facilitative Glucose Transporters. Molecular Biology Intelligence Unit.

[107] Santalucía T., Camps M., Castelló A., Muñoz P., Nuel A., Testar X., Palacin M., Zorzano A. (1992). Developmental Regulation of GLUT-1 (Erythroid/Hep G2) and GLUT-4 (Muscle/Fat) Glucose Transporter Expression in Rat Heart, Skeletal Muscle, and Brown Adipose Tissue. Endocrinology.

[108] Shah H., Gannaban R.B., Haque Z.F., Dehghani F., Kramer A., Bowers F., Ta M., Huynh T., Ramezan M., Maniates A. (2024). BCAAs Acutely Drive Glucose Dysregulation and Insulin Resistance: Role of AgRP Neurons. Nutr. Diabetes.

[109] De la Cruz J.F., Pacunla K.W.M., Hwang S.G. (2022). Low Lysine Stimulates Adipogenesis through ZFP423 Upregulation in Bovine Stromal Vascular Cells. J. Anim. Sci. Technol..

[110] Kovacs P., Hanson R.L., Lee Y.-H., Yang X., Kobes S., Permana P.A., Bogardus C., Baier L.J. (2003). The Role of Insulin Receptor Substrate-1 Gene (IRS1) in Type 2 Diabetes in Pima Indians. Diabetes.

[111] Mackenzie R.W., Elliott B.T. (2014). Akt/PKB Activation and Insulin Signaling: A Novel Insulin Signaling Pathway in the Treatment of Type 2 Diabetes. Diabetes Metab. Syndr. Obes..

[112] Crossland H., Smith K., Idris I., Phillips B.E., Atherton P.J., Wilkinson D.J. (2020). Exploring Mechanistic Links between Extracellular Branched-Chain Amino Acids and Muscle Insulin Resistance: An in Vitro Approach. Am. J. Physiol. Cell. Physiol..

[113] Guo F., Cavener D.R. (2007). The GCN2 eIF2α Kinase Regulates Fatty-Acid Homeostasis in the Liver during Deprivation of an Essential Amino Acid. Cell Metab..

[114] Cheng Y., Meng Q., Wang C., Li H., Huang Z., Chen S., Xiao F., Guo F. (2010). Leucine Deprivation Decreases Fat Mass by Stimulation of Lipolysis in White Adipose Tissue and Upregulation of Uncoupling Protein 1 (UCP1) in Brown Adipose Tissue. Diabetes.

[115] Maida A., Chan J.S.K., Sjøberg K.A., Zota A., Schmoll D., Kiens B., Herzig S., Rose A.J. (2017). Repletion of Branched Chain Amino Acids Reverses mTORC1 Signaling but Not Improved Metabolism during Dietary Protein Dilution. Mol. Metab..

[116] Chen Z., Yang L., Liu Y., Huang P., Song H., Zheng P. (2022). The Potential Function and Clinical Application of FGF21 in Metabolic Diseases. Front. Pharmacol..

[117] Berglund E.D., Li C.Y., Bina H.A., Lynes S.E., Michael M.D., Shanafelt A.B., Kharitonenkov A., Wasserman D.H. (2009). Fibroblast Growth Factor 21 Controls Glycemia via Regulation of Hepatic Glucose Flux and Insulin Sensitivity. Endocrinology.

[118] Kharitonenkov A., Shiyanova T.L., Koester A., Ford A.M., Micanovic R., Galbreath E.J., Sandusky G.E., Hammond L.J., Moyers J.S., Owens R.A. (2005). FGF-21 as a Novel Metabolic Regulator. J. Clin. Investig..

[119] Coskun T., Bina H.A., Schneider M.A., Dunbar J.D., Hu C.C., Chen Y., Moller D.E., Kharitonenkov A. (2008). Fibroblast Growth Factor 21 Corrects Obesity in Mice. Endocrinology.

[120] Sarruf D.A., Thaler J.P., Morton G.J., German J., Fischer J.D., Ogimoto K., Schwartz M.W. (2010). Fibroblast Growth Factor 21 Action in the Brain Increases Energy Expenditure and Insulin Sensitivity in Obese Rats. Diabetes.

[121] Xu J., Lloyd D.J., Hale C., Stanislaus S., Chen M., Sivits G., Vonderfecht S., Hecht R., Li Y.-S., Lindberg R.A. (2009). Fibroblast Growth Factor 21 Reverses Hepatic Steatosis, Increases Energy Expenditure, and Improves Insulin Sensitivity in Diet-Induced Obese Mice. Diabetes.

[122] De Sousa-Coelho A.L., Marrero P.F., Haro D. (2012). Activating Transcription Factor 4-Dependent Induction of FGF21 during Amino Acid Deprivation. Biochem. J..

[123] Richardson N.E., Konon E.N., Schuster H.S., Mitchell A.T., Boyle C., Rodgers A.C., Finke M., Haider L.R., Yu D., Flores V. (2021). Lifelong Restriction of Dietary Branched-Chain Amino Acids Has Sex-Specific Benefits for Frailty and Lifespan in Mice. Nat. Aging.

[124] Laeger T., Henagan T.M., Albarado D.C., Redman L.M., Bray G.A., Noland R.C., Münzberg H., Hutson S.M., Gettys T.W., Schwartz M.W. (2014). FGF21 Is an Endocrine Signal of Protein Restriction. J. Clin. Investig..

[125] Laeger T., Albarado D.C., Burke S.J., Trosclair L., Hedgepeth J.W., Berthoud H.-R., Gettys T.W., Collier J.J., Münzberg H., Morrison C.D. (2016). Metabolic Responses to Dietary Protein Restriction Require an Increase in FGF21 That Is Delayed by the Absence of GCN2. Cell. Rep..

[126] Hill C.M., Laeger T., Dehner M., Albarado D.C., Clarke B., Wanders D., Burke S.J., Collier J.J., Qualls-Creekmore E., Solon-Biet S.M. (2019). FGF21 Signals Protein Status to the Brain and Adaptively Regulates Food Choice and Metabolism. Cell Rep..

[127] Hashimoto O., Tsuchida K., Ushiro Y., Hosoi Y., Hoshi N., Sugino H., Hasegawa Y. (2002). cDNA Cloning and Expression of Human Activin betaE Subunit. Mol. Cell. Endocrinol..

[128] Fang J., Wang S.Q., Smiley E., Bonadio J. (1997). Genes Coding for Mouse Activin Beta C and Beta E Are Closely Linked and Exhibit a Liver-Specific Expression Pattern in Adult Tissues. Biochem. Biophys. Res. Commun..

[129] Namwanje M., Brown C.W. (2016). Activins and Inhibins: Roles in Development, Physiology, and Disease. Cold Spring Harb. Perspect. Biol..

[130] Deli A., Kreidl E., Santifaller S., Trotter B., Seir K., Berger W., Schulte-Hermann R., Rodgarkia-Dara C., Grusch M. (2008). Activins and Activin Antagonists in Hepatocellular Carcinoma. World. J. Gastroenterol..

[131] Hashimoto O., Funaba M., Sekiyama K., Doi S., Shindo D., Satoh R., Itoi H., Oiwa H., Morita M., Suzuki C. (2018). Activin E Controls Energy Homeostasis in Both Brown and White Adipose Tissues as a Hepatokine. Cell. Rep..

[132] Sekiyama K., Ushiro Y., Kurisaki A., Funaba M., Hashimoto O. (2019). Activin E Enhances Insulin Sensitivity and Thermogenesis by Activating Brown/Beige Adipocytes. J. Vet. Med. Sci..

[133] Hashimoto O., Ushiro Y., Sekiyama K., Yamaguchi O., Yoshioka K., Mutoh K., Hasegawa Y. (2006). Impaired Growth of Pancreatic Exocrine Cells in Transgenic Mice Expressing Human Activin betaE Subunit. Biochem. Biophys. Res. Commun..

[134] Hashimoto O., Sekiyama K., Matsuo T., Hasegawa Y. (2009). Implication of Activin E in Glucose Metabolism: Transcriptional Regulation of the Inhibin/Activin betaE Subunit Gene in the Liver. Life Sci..

[135] Hashimoto O., Funaba M. (2011). Activin in Glucose Metabolism. Vitam. Horm..

[136] Griffin J.D., Buxton J.M., Culver J.A., Barnes R., Jordan E.A., White A.R., Flaherty S.E., Bernardo B., Ross T., Bence K.K. (2023). Hepatic Activin E Mediates Liver-Adipose Inter-Organ Communication, Suppressing Adipose Lipolysis in Response to Elevated Serum Fatty Acids. Mol. Metab..

[137] Deaton A.M., Dubey A., Ward L.D., Dornbos P., Flannick J., Yee E., Ticau S., Noetzli L., Parker M.M., AMP-T2D-GENES Consortium (2022). Rare Loss of Function Variants in the Hepatokine Gene INHBE Protect from Abdominal Obesity. Nat. Commun..

