# Supplementary material for: Dietary Insulinogenic Amino Acid Restriction Improves Glucose Metabolism in a Neonatal Piglet Model
Source: Nutrients. 2025 May 15;17(10):1675. doi: 10.3390/nu17101675 (PMC12114165; doi:10.3390/nu17101675)
Supplement: Supplementary file 1 [file nutrients-17-01675-s001.zip › nutrients-3588472-supplementary.pdf]

Supplementary Table S1. The Genes, sequences forward (F) and reverse (R), amplicon size (bp), location on template, and GenBank accession numbers for primers used for reverse transcription qualitative real-time polymerase chain reaction. (RT-qPCR).

Genes[1]	Sequence (5' → 3')	Amplicon length (bp)	Location on template	GenBank accession no.
GCK	F: CCGACTTCCTGGACAAGCAT	238	1040–1059	XM_003134883.2
	R: ATCGTGGCCACAGTGTCATT		1258–1277	
HK2	F: ATGGAGGAGATGCGGAATGT	184	2063–2082	NM_001122987.1
	R: TGCCGCTGATCATTTTCTCG		2227–2246	
GLUT2	F: GGTTCATGGTGGCCGAGTT	83	1260–1278	NM_001097417.1
	R: ATTGCGGGTCCAGTTGC		1326–1342	
PEPCK2	F: CTGGGAAGGCATTGATCAGC	110	1336–1335	NM_001161753.1
	R: AGCGAGAGTTAGGATGTACA		1426–1445	
PFKL	F: ACTCCCTTCGACCGGAACTA	197	2119–2138	XM_021071510.1
	R: TGCTCAAAGTCGGTGTCCTA		2296–2315	
PKLR	F: CCCACTGAAGTCACCGCTAT	288	1357–1376	XM_021089721.1
	R: GAGGAAGCCACGGAGTTTT		1626–1644	
G6PC	F: TGAACGTCTGTCTGTCACGA	137	491–510	NM_001113445.1
	R: ATACTTCTTGAGGCTGGCGT		608–627	
GLUT1	F: GGAGATGAAGGAGGAGAGCC	150	982–1001	XM_021096908.1
	R: TAGAAAACCGCGTTGATGCC		1112–1131	
PC	F: GGACTTCACTGCCACCTTTG	92	3063–3082	NM_214349.1
	R: GCTCCACCTCAAACTCCTCT		3135–3154	
AKT1	F: AGAAGCTCTTCGAGCTCATCCTCA	148	1102–1125	NM_001159776.1
	R: TGCATGATCTCCTTGGCATCCTCA		1226–1249	
IRS1	F: AGGAAGTTTGGCAGGTGATCCTGA	200	519–542	NM_001244489.1
	R: ACGGCCCACTTCGATGAAGAAGAA		658–681	
S6K	F: TCCAATACGACAGCCGAACT	163	1626–1645	XM_003131671.5
	R: TCACCTTGCAGGATGCTCAC		1769–1788	
IR	F: TTCACTGGCAATCGCATTGAGCTG	137	2913–2936	XM_021083940.1
	R: TCATGGGTCACAGGGCCAATGATA		3026–3049	
GYS2	F: AATTCTGTGGGAAGCCCAGC	92	100–119	NM_001195511.1
	R: GCAGGGATTCAGAGCTGGTA		172–191	
PYGL	F: ATCGGGGAGGACTACGTGAA	79	1522–1541	NM_001123172.1
	R: CTCGGAGGAAGACATCGTCG		1581–1600	
GSK-3α	F: AGCTGATCTTTGGAGCCACC	170	889–908	NM_001315708.1
	R: TCGAATCTGTTCCCGGGTTG		1039–1058	
GSK-3β	F: CGAGACACACCTGCACTCTT	186	1096–1115	NM_001128443.1
	R: TGACGCAGAAGCGGTGTTAT		1281–1262	
GLUT4	F: GAAGGAAGAAGGCAATGCTG	300	354–373	NM_001128433.1
	R: CCAGGAGTAGTGGCCATAGG		634–653	
PFKM	F: TCATCATTGTGGCTGAGGCT	160	877–896	NM_001044550.1
	R: AGGATTCTGTCAAAGGCCGA		1017–1036	
CPT1α	F: CAAGATGGGCATGAACGCTG	145	1406–1425	NM_001129805.1

	R: TGGAATGTTGGGGTTGGTGT		1531–1550	
CPT1β	F: TTCAACACTACACGCATCCC	117	1219–1238	XM_021091195
	R: GCCCTCATAGAGCCAGACC		1317–1335	
ACC	F: ATGTTTCGGCAGTCCCTGAT	133	4870–4889	NM_001114269.1
	R: TGTGGACCAGCTGACCTTGA		4983–5002	
FAS	F: CTGCTGAAGCCTAACTCCTCG	207	584–604	NM_001099930.1
	R: TTGCTCCTTGGAACCGTCTG		771–790	
HSL	F: GCTCCCATCGTCAAGAATC	265	2043–2061	NM_214315.3
	R: TAAAGCGAATGCGGTCC		2291–2307	
SREBP1	F: CGGACGGCTCACAATGC	114	986–1002	NM_214157.1
	R: GACGGCGGATTTATTCAGCTT		1079–1099	
HADH	F: GCCATCGTGGAGAACCTGAA	159	461–480	NM_214331.1
	R: GAAATGGAGCCCGGCAAATC		600–619	
CD36	F: CTGGTGCTGTCATTGGAGCAGT	161	443–464	NM_001044622.1
	R: CTGTCTGTAAACTTCCGTGCCTGTT		579–603	
LPL	F: CCCTATACAAGAGGGAACCGGAT	138	448–470	NM_214286.1
	R: CCGCCATCCAGTCGATAAACGT		564–580	
FGF-21	F: CCTGAAGCCAGGGGTCATTC	85	382–401	NM_001163410.1
	R: CGATCCGTACAGTCTCCCGT		447–466	
KLB	F: ATCGACGACCAGTCTCTGGA	231	2812–2831	XM_003482367.4
	R: AGGGAAGCCATTGTTGCTGA		3023–3042	
PPARα	F: GGCACTGAACATCGAATGTAGAAT	80	319–352	NM_001044526.1
	R: TGCAACCTTCACAGGCATGA		389–418	
ATF4	F: GGGCTGAAGAGAGCTTAGGG	69	1068–1087	XM_021090887.1
	R: ACCCATGAGGTTTGAAGTGC		1117–1136	
GCN2	F: GAAATGCTGGAAAGGCAGGC	77	526–545	XM_021097873.1
	R: CGTTGCTCCTGCTCCTCTTT		583–602	
SIRT1	F: GAAACAATGGGCCGGGCTTA	550	296–315	NM_001145750.2
	R: AGGCGTGCATAAATGCCATC		826–845	
PGC-1α	F: TTCCGTATCACCACCCAAAT	137	1673–1692	NM_213963.2
	R: ATCTACTGCCTGGGGACCTT		1790–1809	
EIF2α	F: CTGTACCTGGTGAACGGACC	188	250–269	XM_021069600.1
	R: GCCTTTGGAAGGTCGAAGGA		418–437	
IRE1α	F: GAGTCCGCAGCAGGTG	65	534–549	NM_001142836.1
	R: CCGTCAGAATCCATGGGG		581–598	
PERK	F: ACTACAAGCGGGAAAGGAGC	115	1772–1791	XM_003124925.4
	R: CACCAGTGCAAAAGGAGCAC		1867–1886	
ChREBP	F: GCCGTCATCTTGGAGGGGAA	95	371–390	XM_021086329.1
	R: CGGAGCCGCTTCTTGTAGTA		446–465	
NUPR1	F: CAGCACGGACCTGAAACAGA	82	966–985	NC_010445.4
	R: TCCTTTCCTAGCAGAGGGACC		1026–1047	
INHBE	F: GGCTACACTTGAGCAGTCGT	197	185–204	XM_003126320.4
	R: GGACCGAGGAGTAGACAGGT		362–381	
MAPK14	F: ACAAGACAATCTGGGAGGTA	116	480–499	XM_013977842.2
	R: CACTGCAACACGTAACCC		578–595	

JNK1	F: AACTCTTTGACGCTGCTTGC	106	4291–4310	XM_021073087.1
	R: TGAAGCACTGTGCCTTTACC		4377–4396	
JNK2	F: ACCCCTTGAAGGTTGTCGAT	164	1462–1481	XM_021084317.1
	R: ACAACTGAGTAGGGGCAAGG		1606–1625	
PRKCα	F: CAGATCCTTATGTGAAGCTGAAACTT	80	575–600	XM_021066740.1
	R: TGTAGAGCGGATGGTCTTGGTT		633–654	
PRKCβ	F: CTGTATGAAATGTTGGCTGGGC	254	2147–2168	XM_021086460.1
	R: GGCTGAATCTCTTTGCGTTCG		2380–2400	
PRKCγ	F: AAAGGCAGTTTTGGGAAGG	100	2857–2875	XM_021094907.1
	R: CGTCATCCTGGACAATCACG		2937–2956	
β-Actin	F: CTGCGGCATCCACGAAACT	147	944–962	XM_003124280.5
	R: AGGGCCGTGATCTCCTTCTC		1071–1090	

[1] GCK = glucokinase; GLUT 2 = glucose transporter 2; HK2 = hexokinase 2; PEPCK2 = phosphoenolpyruvate carboxykinase 2; PFKL = phosphofructokinase, liver type; PKLR = pyruvate kinase, liver and RBC; G6PC = glucose-6-phosphatase catalytic; GLUT1 = glucose transporter 1; PC = pyruvate carboxylase; AKT1 = protein kinase B; IRS1 = insulin receptor substrate 1; S6K = ribosomal protein S6 kinase B1; IR = insulin receptor; GYS2 = glycogen synthase 2; PYGL = glycogen phosphorylase, liver form; GSK-3α = glycogen synthase kinase 3α; GSK-3β = glycogen synthase kinase 3β; GLUT4 = glucose transporter 4; PFKM = phosphofructokinase, muscle type; CPT1α = carnitine palmitoyltransferase 1α; CPT1β = carnitine palmitoyltransferase 1β; ACC = acetyl-CoA carboxylase; FAS = fatty acid synthase; HSL = hormone-sensitive lipase; SREBP1 = sterol regulatory element-binding protein 1; HADH = hydroxyacyl-CoA dehydrogenase; CD36 = cluster of differentiation 36; LPL = lipoprotein lipase; FGF-21 = fibroblast growth factor 21; KLB = β-Klotho; PPARα = peroxisome proliferator activated receptor α; ATF4 = activating transcription factor 4; GCN2 = general control nonderepressible 2; SIRT1 = sirtuin 1; PGC-1α = peroxisome proliferator-activated receptor gamma coactivator-1α; EIF2α = eukaryotic translation initiation factor 2α; IRE1α = inositol-requiring enzyme type 1α; PERK = EIF2α kinase 3; ChREBP = carbohydrate-responsive element-binding protein; NUPR1 = nuclear protein 1; INHBE = inhibin subunit beta E; MAPK14 = p38α mitogen-activated protein kinase; JNK1 = mitogen-activated protein kinase 8 ; JNK2 = mitogen-activated protein kinase 9; PRKCα = protein kinase C alpha; PRKCβ = protein kinase C beta; PRKCγ = protein kinase C gamma; β-Actin = Beta-actin.

Primer sequences were obtained from other sources or designed in-house as follows: GCK, GLUT 2, PFK and PKLR (Xie et al., 2016); HK2, PEPCK2, G6PC, GLUT1, PC and PFKM (He et al., 2017); AKT1, IRS1 and IR (Kim et al., 2014); S6K (Zhao et al., 2019); GYS2, PYGL, GSK-3α and GSK-3β (Ying et al., 2017); GLUT4 (Cervantes et al., 2016); CPT1α (Zhou et al., 2016); CPT1β (He et al., 2012); ACC and FAS (Chen et al., 2014); HSL (Fang et al., 2014); SREBP1 (Duran-Montgé et al., 2009); HADH (Zhou et al., 2019); LPL (Espinoza et al., 2020); FGF-21 (Hu et al., 2022a); KLB (Gavaldà-Navarro et al., 2018); ATF4 (Merz et al., 2020); PGC-1α: (Jin et al., 2017); GCN2 and SIRT1 (Niu et al., 2020); CD36 (Li et al., 2017); PPARα (Weber et al., 2008); IRE1α (Zhong et al., 2022); PERK (Zheng et al., 2021); ChREBP (Vitali et al., 2018); MAPK14 (Hu et al., 2022b); JNK2 (Chomwisarutkun et al., 2012); PRKCα (Guo et al., 2003); PRKCβ and PRKCγ (Zorilla et al., 2009); β-Actin (Yin et al., 2015); NUPR1, JNK1, EIF2α and INHBE (In-house).

Supplementary Table S2. Primary and secondary antibodies for immunoblotting, host, dilution, and supplier.

Antibodies	Host	Dilution	Vendor
Anti-FGF-21[1]	Rabbit	1:1000	Abcam, Cambridge, MA, #ab171941
Anti-GAPDH [6C5] (HRP)	Mouse	1:1000	Abcam, Cambridge, MA, #ab105428
Anti-Rabbit IgG H&L (HRP)	Goat	1:2000	Abcam, Cambridge, MA, #ab205718

[1]FGF-21 = fibroblast growth factor 21.

Supplementary Table S3. Effect of insulinogenic amino acid restriction on dry matter intake and growth parameters of neonatal pigs.

**Measurements**	**Diets[1]**				
	**NR**	**R50**	**R75**	**SEM[2]**	***P*-value**
Initial body weight, g	2280	2280	2290	82.45	0.99
Final body weight, g	3250	3140	3050	140.95	0.83
ADG[3], g/day	43.91	40.91	41.92	3.93	0.96
ADMI[3], g/day	131.40	176.31	184.71	13.44	0.21
CDMI[3], g	2688.75	3775.24	4002.82*	261.76	0.07
ADPI[3], g/day	29.47	34.36	38.84	2.56	0.29
G:F[3], g/g	0.31	0.20	0.25	0.02	0.16
G:P[3], g/g	1.46	1.30	1.16	0.11	0.50
Wither height, cm	23.57	22.17	22.33	0.46	0.44
Body length, cm	30.38	29.17	28.56#	0.38	0.11
Heart girth, cm	34.78	34.33	34.33	0.46	0.90

[1] NR: basal diet without restricted insulinogenic amino acid (IAA); R50: basal diet with 50% restricted IAA; R75: basal diet with 75% restricted IAA. The values are the means. n=11 for NR and R75, and n=10 for R50. $^{\#}P \leq 0.1$ vs. NR and $^{*}P \leq 0.05$ vs. NR.

[2]SEM: standard errors of means

[3]ADG: average daily gain; ADMI: average dry matter intake; CDMI: cumulative dry matter intake; ADPI: average daily protein intake; G:F: gain:feed; G:P: gain:protein.

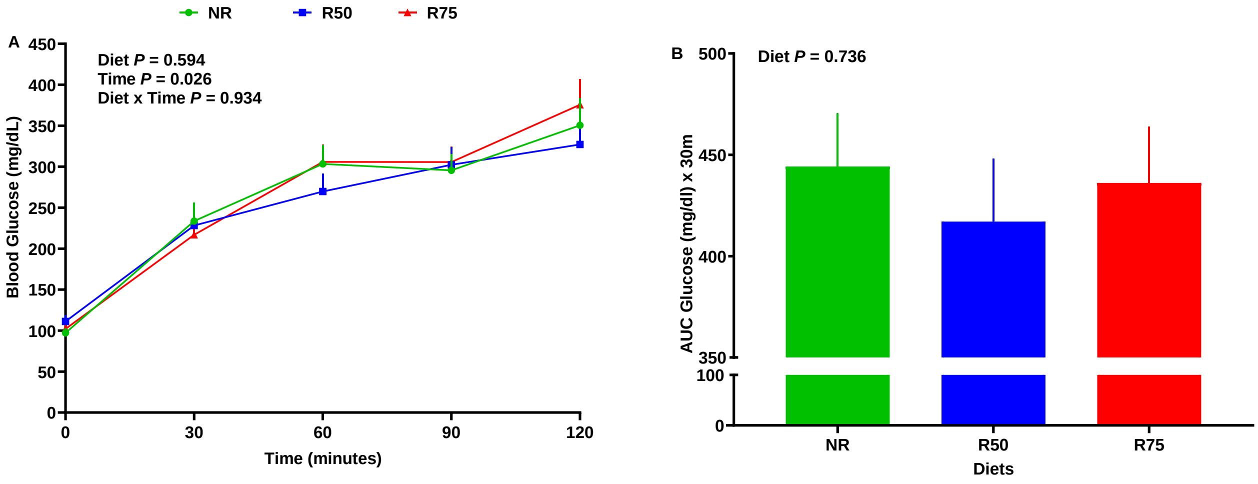

Supplementary Figure S1. Effect of insulinogenic amino acids restriction (IAA) on glucose tolerance. (A) blood glucose and (B) area under the curve (AUC) for blood glucose after a meal test challenge in neonatal pigs. NR: basal diet without restricted IAA; R50: basal diet with 50% restricted IAA; R75: basal diet with 75% restricted IAA. The values are the means ± SE. *n*=9 for CON, *n*=5-7 for 50R, and *n*=7-9 for 75R.

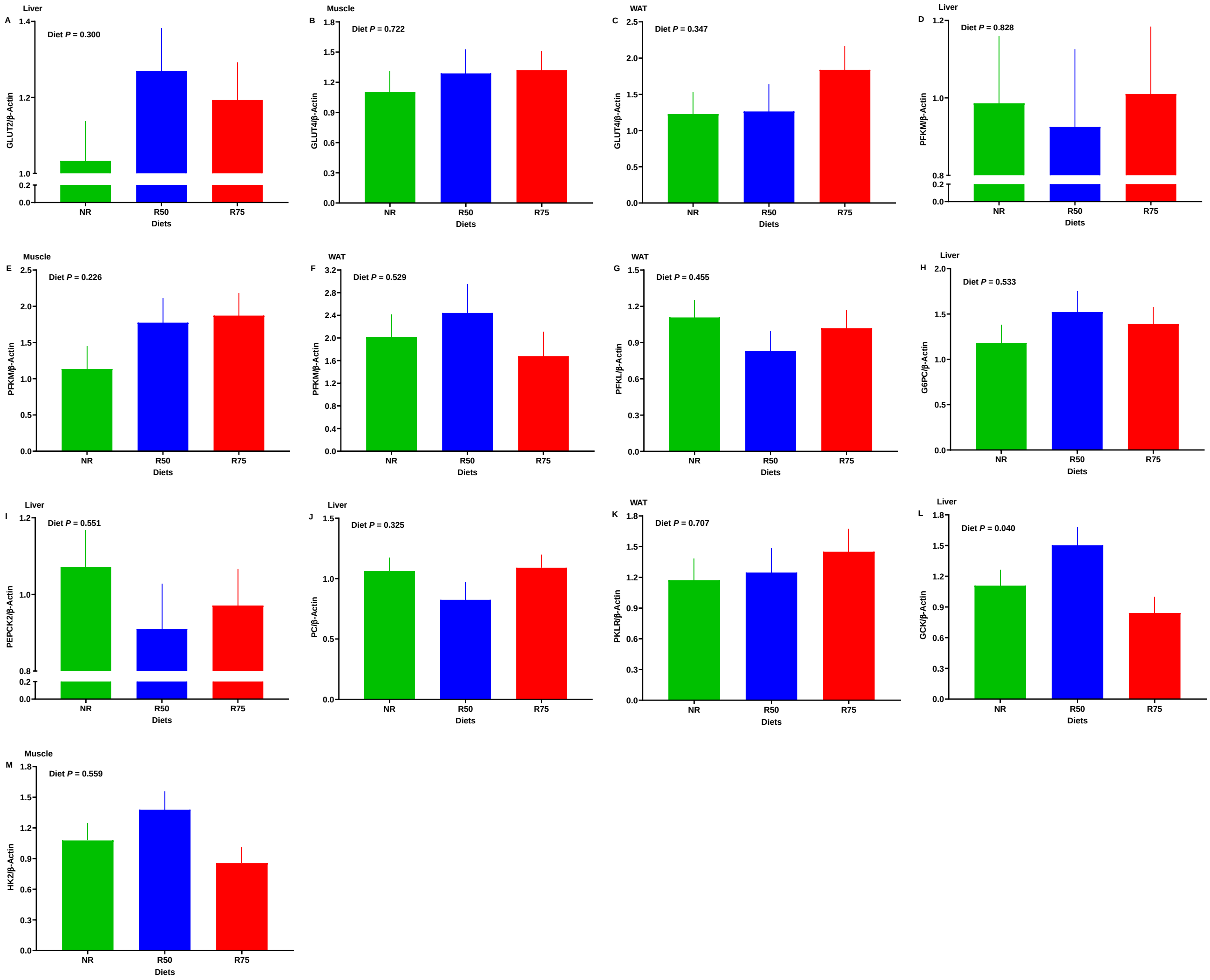

Supplementary Figure S2. Effect of insulinogenic amino acids (IAA) restriction on mRNA abundance of genes involved in glucose transport and metabolism in the liver, white adipose tissue (WAT), and skeletal muscle of neonatal pigs. (A) liver glucose transporter 2 (GLUT2), (B) skeletal muscle glucose transporter 4 (GLUT4), (C) WAT GLUT4, (D) liver phosphofructokinase, muscle type (PFKM), (E) skeletal muscle PFKM, (F) WAT PFKM, (G) WAT phosphofructokinase, liver type (PFKL), (H) liver glucose-6-phosphatase catalytic (G6PC), (I) liver phosphoenolpyruvate carboxykinase 2 (PEPCK2), (J) liver pyruvate carboxylase (PC), (K) WAT pyruvate kinase, liver and RBC (PKLR), (L) liver glucokinase (GCK), and (M) skeletal muscle hexokinase 2 (HK2). NR: basal diet without restricted IAA; R50: basal diet with 50% restricted IAA; R75: basal diet with 75% restricted IAA. The values are the means ± SE. *n*=8-9 for NR, *n*=5-7 for R50, and *n*=7-9 for R75.

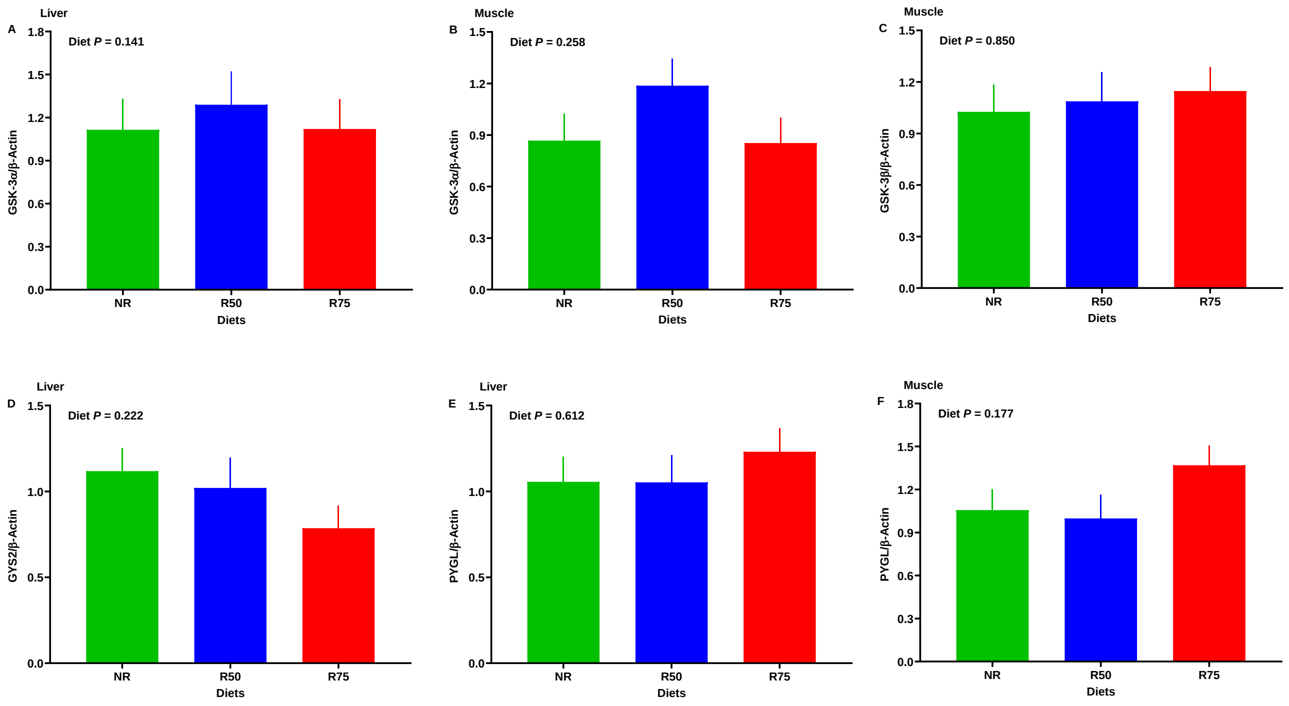

Supplementary Figure S3. Effect of insulinogenic amino acids (IAA) restriction on mRNA abundance of genes involved in glycogen metabolism in the liver and skeletal muscle of neonatal pigs. (A) liver glycogen synthase kinase 3α (GSK-3α), (B) skeletal muscle GSK-3α, (C) skeletal muscle glycogen synthase kinase 3β (GSK-3β), (D) liver glycogen synthase 2 (GYS2), (E) liver glycogen phosphorylase (PYGL), and (F) skeletal muscle PYGL. NR: basal diet without restricted IAA; R50: basal diet with 50% restricted IAA; R75: basal diet with 75% restricted IAA. The values are the means ± SE. *n*=7-9 for NR, *n*=5-7 for R50, and *n*=8-9 for R75.

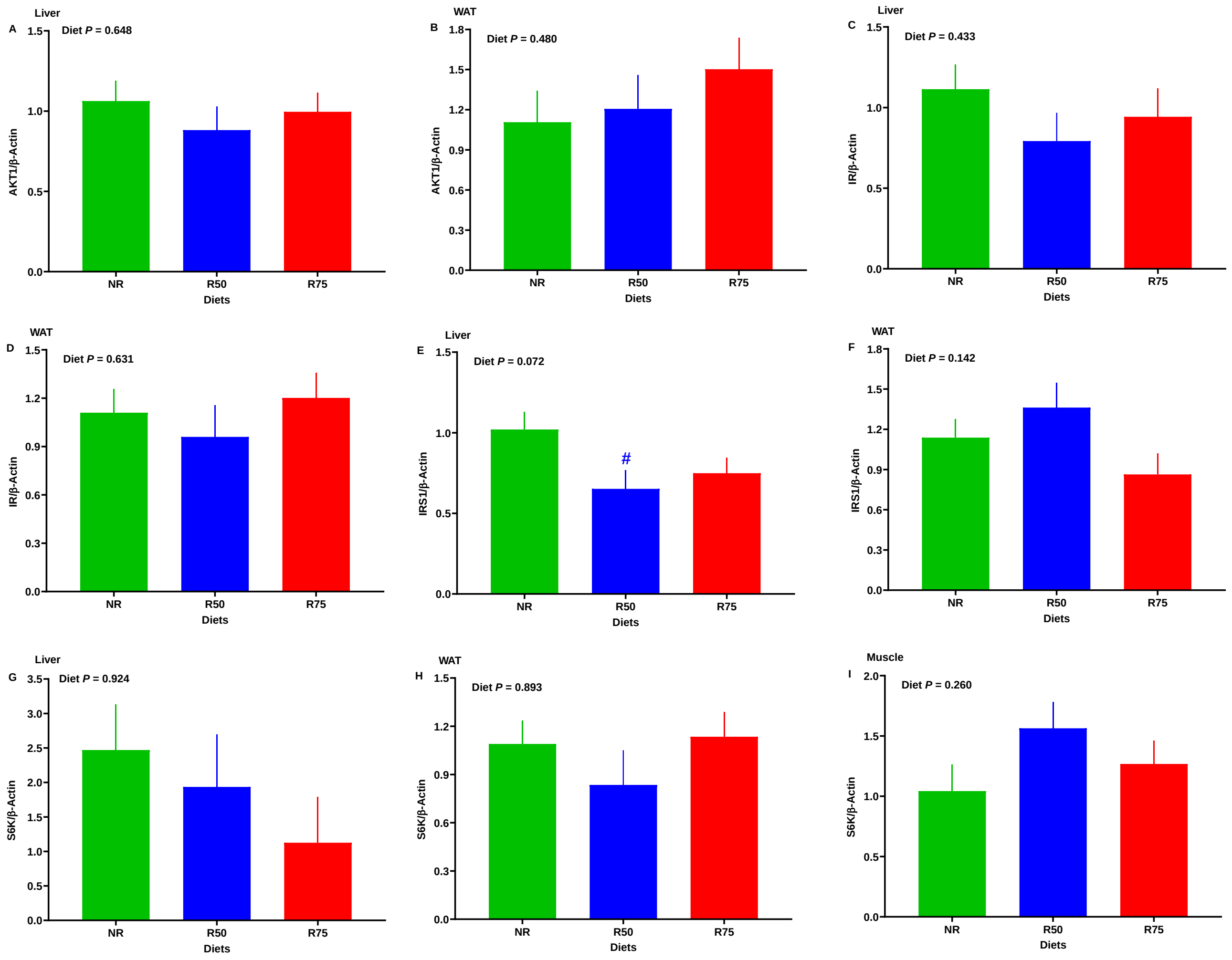

Supplementary Figure S4. Effect of insulinogenic amino acids (IAA) restriction on mRNA abundance of genes involved in insulin signaling in the liver, white adipose tissue (WAT), and skeletal muscle of neonatal pigs. (A) liver protein kinase B (AKT1), (B) WAT AKT1, (C) liver insulin receptor (IR), (D) WAT IR, (E) liver insulin receptor substrate 1 (IRS1), (F) WAT IRS1, (G) liver ribosomal protein S6 kinase B1 (S6K), (H) WAT S6K, and (I) skeletal muscle S6K. NR: basal diet without restricted insulinogenic IAA; R50: basal diet with 50% restricted IAA; R75: basal diet with 75% restricted IAA. The values are the means ± SE. *n*=7-9 for NR, *n*=4-7 for R50, and *n*=6-9 for R75. $^{\#}P \leq 0.1$ vs. NR.

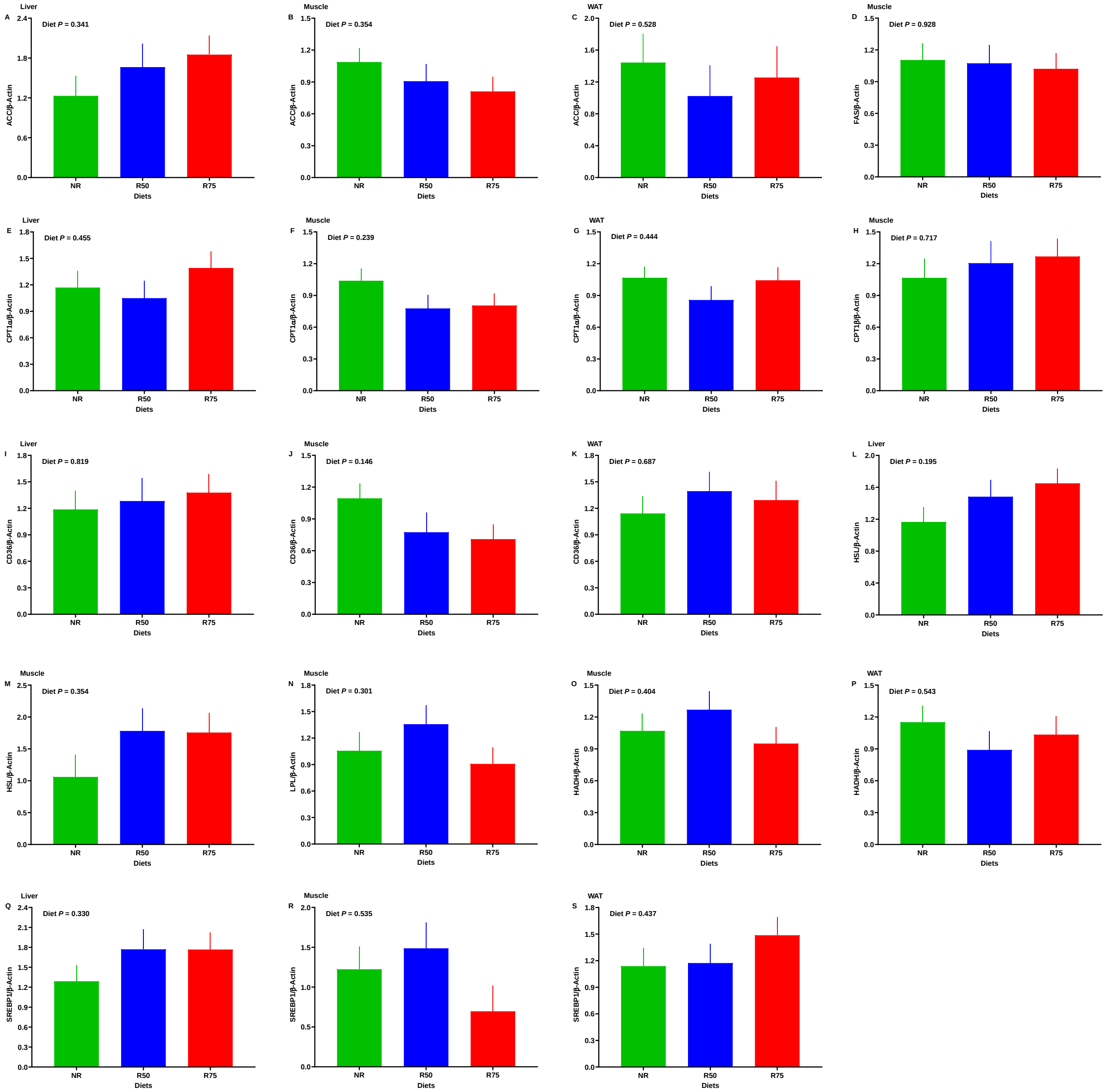

Supplementary Figure S5. Effect of insulinogenic amino acids (IAA) restriction on mRNA abundance of genes involved in lipid metabolism in the liver, white adipose tissue (WAT), and skeletal muscle of neonatal pigs. (A) liver acetyl-CoA carboxylase (ACC), (B) skeletal muscle ACC, (C) WAT ACC, (D) skeletal muscle fatty acid synthase (FAS), (E) liver carnitine palmitoyltransferase 1α (CPT1α), (F) skeletal muscle CPT1α, (G) WAT CPT1α, (H) skeletal muscle carnitine palmitoyltransferase 1β (CPT1β), (I) liver cluster of differentiation 36 molecule (CD36), (J) skeletal muscle CD36, (K) WAT CD36, (L) liver hormone-sensitive lipase (HSL), (M) skeletal muscle HSL, (N) skeletal muscle lipoprotein lipase (LPL), (O) skeletal muscle hydroxyacyl-CoA dehydrogenase (HADH), (P) WAT HADH, (Q) liver sterol regulatory element-binding protein 1 (SREBP1), (R) skeletal muscle SREBP1, (S) WAT SREBP1. NR: basal diet without restricted IAA; R50: basal diet with 50% restricted IAA; R75: basal diet with 75% restricted IAA. The values are the means ± SE. *n*=7-9 for NR, *n*=5-7 for R50, and *n*=7-9 for R75.

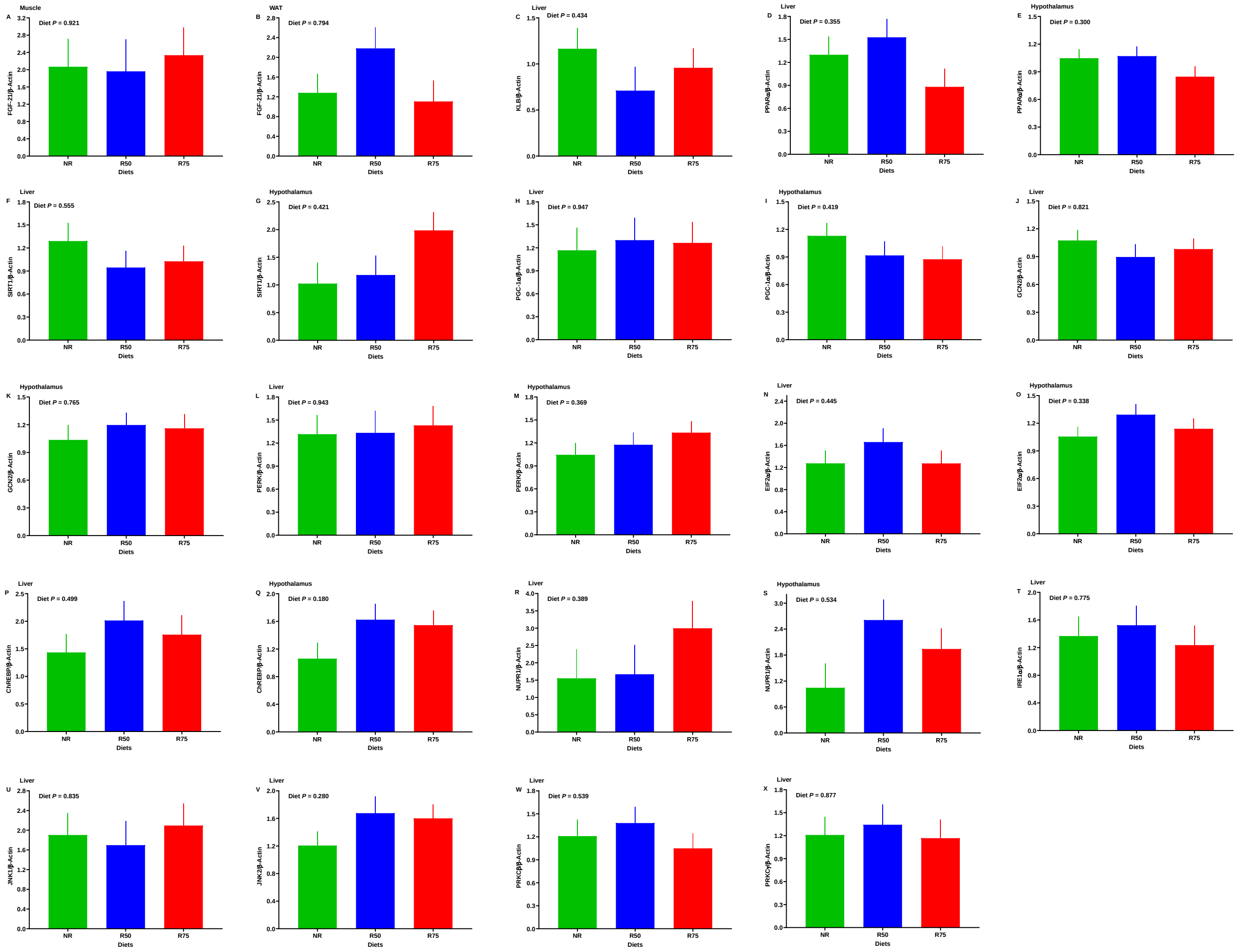

Supplementary Figure S6. Effect of insulinogenic amino acids (IAA) restriction on mRNA abundance of genes involved in FGF-21 pathway in the liver, white adipose tissue (WAT), and hypothalamus of neonatal pigs. (A) skeletal muscle fibroblast growth factor 21 (FGF-21), (B) WAT FGF-21, (C) liver β-Klotho (KLB), (D) liver peroxisome proliferator activated receptor α (PPARα), (E) hypothalamus PPARα, (F) liver sirtuin 1 (SIRT1), (G) hypothalamus SIRT1, (H) liver peroxisome proliferator-activated receptor gamma coactivator-1α (PGC-1α), (I) hypothalamus PGC-1α, (J) liver general control nonderepressible 2 (GCN2), (K) hypothalamus GCN2, (L) liver eukaryotic translation initiation factor 2α kinase 3 (PERK), (M) hypothalamus PERK, (N) liver eukaryotic translation initiation factor 2A (EIF2A), (O) hypothalamus EIF2A, (P) liver carbohydrate-responsive element-binding protein (ChREBP), (Q) hypothalamus ChREBP, (R) liver nuclear protein 1 (NUPR1), (S) hypothalamus NUPR1, (T) liver inositol-requiring enzyme type 1α (IRE1α), (U) liver mitogen-activated protein kinase 8 (JNK1), (V) liver mitogen-activated protein kinase 9 (JNK2), (W) liver protein kinase C beta (PRKCβ), and (X) liver protein kinase C gamma (PRKCγ). NR: basal diet without restricted IAA; R50: basal diet with 50% restricted IAA; R75: basal diet with 75% restricted IAA. The values are the means ± SE. *n*=5-9 for NR, *n*=5-7 for R50, and *n*=6-8 for R75.
